# COVID-19 and distortions in urban food market in India

**DOI:** 10.1007/s41775-022-00130-3

**Published:** 2022-05-30

**Authors:** Pallavi Rajkhowa, Lukas Kornher

**Affiliations:** Center for Development Research (ZEF), University of Bonn, Bonn, Germany

**Keywords:** COVID-19, Food prices, Market distortion, Price dispersion, India

## Abstract

In this paper, we study the effects of the spread of COVID-19 on retail and wholesale prices of urban markets in India, as well as price distortion between markets and the mark-up between retail and wholesale prices. Using fixed-effects panel regression models, we find that with the spread of COVID-19, prices increased for commodities with longer shelf-life such as pulses and processed items, while prices of vegetables such as onions and tomatoes declined substantially at the onset of the pandemic. Further, market distortions increased significantly for most commodities. Pulses experienced large price distortions between markets as well as mark-ups between retail and wholesale prices. We, however, do not see any major price distortions in the market for rice and wheat, which are controlled by Government’s minimum support prices.

**Supplementary Information:**

The online version contains supplementary material available at 10.1007/s41775-022-00130-3.

## Introduction

The world experienced one of its worst health crises with the onset of the COVID-19 pandemic in early 2020. Governments in high-income and low- and middle-income countries (LMIC) imposed stringent non-pharmaceutical measures to prevent the spread of the virus and minimize mortality and health damages at a time when vaccines were unavailable and testing capacity was severely constrained (Ruan et al., 2021). These measures included restrictions on travel, the closing of schools, prohibiting gatherings, and closure of workplaces, informal food markets, shops, and public transportations. Such negative shocks have had severe consequences on all economic activities, including the smooth operation of food supply networks—caused by distortions in consumer food demand and supply-side bottlenecks—particularly in low-and-middle-income countries where value chains are less structured (Akter, 2020; Mahajan & Tomar, 2021; Ruan et al., 2021).

The increase in food costs due to COVID-19-related disruptions in the supply chain could have severe consequences on food access and the quality of diet (Devereux et al., 2020; Harris et al., 2020). Higher food prices hurt the poorest section of the society most, especially at a time when many have lost their livelihoods due to the closure of non-essential activities in the economy. Further, the reduced purchasing power of poorer households, leads to substitution for less desirable but less expensive products, potentially lowering prices in these value chains (Hirvonen et al., 2021).

India went into one of the most severe national lockdowns from 24 to 31st May 2020 (Lowe et al., 2021; Mahajan & Tomar, 2021). Although the national lockdown was imposed at the end of the first quarter of 2020, few states^1^ were already imposing restrictions on movements and public gatherings before the all-India lockdown.^2^ In regions where COVID-19 caseloads were spreading rapidly, there was strict vigilance by authorities in the adherence to the restrictions on consumer mobility, gatherings, logistics, and business activities, as well as community surveillance to isolate infected cases in containment zones. In such situations, risk-averse individuals are likely to engage in voluntary precautionary measures irrespective of Government rules. Thus, constant information through media outlets such as newspapers, social network platforms, and media channels about the spread and the risk of COVID-19 is likely to have affected regular activities during the pandemic—such as going to the market or using public transportation—in different ways depending on people’s risk-taking abilities (Chan et al., 2020).

Globally there is a growing body of literature analyzing the initial effects of COVID-19-related policy restrictions on food markets. Using data from European countries, Akter (2020) reveals that COVID-19-related "stay at home" regulations increased food prices in March 2020, with meat, fish, seafood, and vegetables seeing the largest increase. Ruan et al. (2021) study the implications of the Chinese lockdown policies on vegetable prices, specifically on Chinese cabbage. They find that price and price dispersion increased due to the stringent measures in the initial weeks but came down eventually when the lockdown measures were largely removed. Dietrich et al. (2021) find that stringent policy responses increased food prices for integrated markets but not for segmented markets. In the Indian context as well, several studies have analyzed the immediate effects of COVID-19-related disruptions on retail and wholesale markets. For instance, Lowe et al. (2021) evaluate the early effects of the pandemic on arrivals and prices of food items traded in wholesale markets. Their study finds that 3 weeks following the first national lockdown, food arrivals in wholesale markets dropped significantly while food prices increased moderately, but after 6 weeks of lockdown markets stabilized and volumes and prices had fully recovered. Similarly, Narayanan and Saha (2021) analyze the trends in food prices in retail and wholesale markets in the first month of the national lockdown and they find that many commodities witnessed a rise in prices just after the lockdown. Mahajan and Tomar (2021) analyze the impact of the first lockdown on ‘online’ retail prices and availability of perishable and non-perishable commodities, as well as arrivals of fruits and vegetables in agricultural wholesale markets. They find that in the immediate aftermath of the first national lockdown, product availability in online markets and arrivals in wholesale markets declined but there was little impact on online prices. Further, Varshney et al. (2020) evaluate the impact of COVID-19 on the wholesale quantities and prices of wheat, tomato, and onion over a relatively long period (over 3 months), thus going beyond the first lockdown. They find that for wheat, there was a dramatic decrease in the market arrivals in the first two phases of the lockdown compared to the year 2019, but the market arrivals were recovered in the later lockdown phases. Mirroring the disruption of the market arrivals, the prices of wheat were higher in the first lockdown phase as compared to prices in 2019 but eventually, prices fell during the later lockdown phases.

In this paper using data from urban markets in India, we study the effects of the spread of COVID-19 on market prices, as well as market distortions for 15 commodities ranging from perishable items such as vegetables to items with longer shelf-life such as cereals, pulses, and processed food items. It is expected that markets located in regions where COVID-19 caseloads increased rapidly are likely to have experienced larger disruptions in their supply chains and changes in consumer demand. Further, disruptions in upstream food supply chains are likely to increase the wedge in wholesale and retail prices, and disruptions in mobility can increase spatial price dispersion between markets.

Several studies have shown that prices in general increased for most commodities and the increase was largest for perishables (Akter, 2020; Ruan et al., 2021). However, this pattern could be different in the Indian context because the timing of the spread of COVID-19 and related interventions coincided with the harvest season for perishables such as onions, tomatoes, and potatoes (Government of India, 2020a, 2020b, 2020c) which could have created an oversupply in production hubs and resulted in distressed sales due to the inability to bring the product to the market. Bairagi et al. (2022) using high-frequency phone survey data by the World Bank find that prices of storable foodstuffs, such as wheat flour and rice, increased while prices of onions declined in India due to the nationwide lockdown. In line with this finding, we expect that demand for storable with longer shelf-life could have increased due to increased consumer demand—due to substitution of commodities with short shelf-life such as vegetables towards more storable items such as pulses and processed items—as well as the ability of traders to hoard these items for sales later. In addition to that, non-perishable products can be traded over a longer distance, and therefore, supply chain disruptions could have a greater effect on prices for storable items. In contrast, perishable items, especially those that were harvested just about the time of the initial phase of national lockdown, could have experienced a large decline in institutional demand due to two main reasons. The first is the closure of hotels, restaurants, offices, and street food vendors. Second is a decline in household consumer demand. According to Engel’s Law, the share of expenditures for staple foods is inversely related to total household expenditures. In consequence, reduced purchasing power could cause the substitution of vegetable products for staples (Hirvonen et al., 2021). Therefore, we expect the effects on food prices to vary depending on the type of commodity.

This paper complements Varshney et al.'s (2020) study in terms of the time frame of analysis, as well as the existing literature on short-term price effects. Specifically, we add to the growing body of literature on COVID-19 and its implications on food and agricultural markets in five key ways. First, we analyze the effects of the spread of COVID-19 on both retail and wholesale food markets. Second, while the studies cited on this topic have looked at the effects on market prices, we also analyze the impacts on mark-ups between retail and wholesale prices. Third, we analyze the effects of the spread of COVID-19 on spatial price dispersions between markets. Fourth, we analyze the price effects on a range of commodities including perishable vegetables, and items with longer shelf-life such as cereals, pulses, and processed food items to get a better understanding of the effects depending on the type and nature of the commodity. Last, and most importantly, all the papers cited on this topic use some version of a difference-in-differences or event study approach as their estimation strategy; however, most of these papers do not control for inter-related time-varying factors or consider trends and seasonality which are important components of time-series data. We, therefore, build on their empirical strategy by incorporating time-varying factors such as rainfall, transportation costs, and economic activity which are likely to affect market prices and account for issues such as seasonality, trends, the spatial and temporal correlation between, and across markets.

Using fixed-effect panel regression models, we find a statistically significant association between the spread of COVID-19 and retail and wholesale prices as well as market distortions. In general, we find that prices increased for commodities with longer shelf-life, while perishable commodities such as onions and tomatoes declined substantially. The implementation of COVID-19-related restrictions coincided with the harvest of ‘late Kharif’ and ‘Rabi’ season crops, which could have caused an oversupply in production hubs and therefore lowered prices. We also find that, for most commodities, market distortions had increased significantly. The largest price distortions between markets as well as the mark-ups between retail and wholesale prices were witnessed by pulses, but we do not see any significant price distortions for rice and wheat which are controlled by Government’s minimum support prices. However, we do find that the decline in retail and wholesale prices of onions due to an oversupply reduced existing price distortions in markets. The same was not true for tomatoes, wherein despite the decline in overall prices, market distortions increased.

The paper proceeds as follows. Section 2 gives a background of the severity of the COVID-19 infection in India and gives an overview of the different pathways through which we expect the spread of the pandemic to affect market outcomes. Section 3 describes the data, the main econometric specifications, and the related robustness check used for our analysis. Section 4 discusses the results and Sect. 5 summarizes the conclusions of the paper.

## Background

### Spread of COVID-19 in India and containment measures of the government

**Fig. 1:**
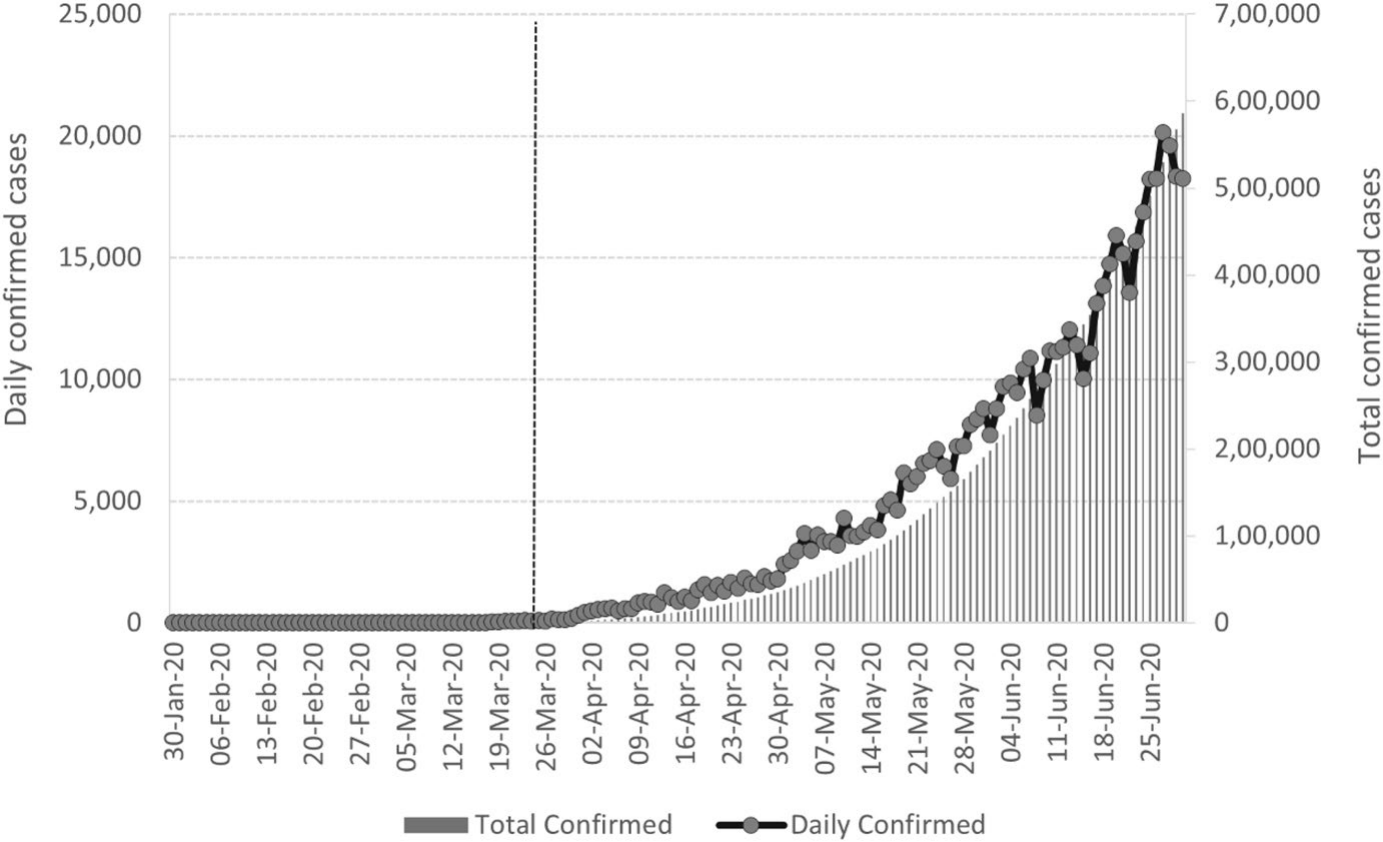
Incidence of COVID-19 cases in India (up to June 2020). Source: COVID-19 India tracker. Data accessed 13.05.2021. The dotted line shows the date of the first national lockdown

India reported its first COVID-19 case on January 30th, 2020, in Kerela, located in southern India. On 3rd February, the Kerela Government declared the coronavirus a state emergency. By mid-March, infection rates across India accelerated to over 100 confirmed cases and over 1000 by the end of March (Fig. 1). On March 22nd, a 14-h voluntary “Janta-curfew”^3^ was implemented, followed by a full lockdown from 24th March for 3 weeks. The national lockdown thereafter was extended for another 21 days, till May 3rd, and then extended again till May 31st. The lockdown was imposed at a time when confirmed COVID-19 cases were around 500 among India’s 1.3 billion people. Also, all activities were shut down across all regions at the same time, even when the COVID-19 contagion was limited in certain geographical areas (Mahajan & Tomar, 2021). Some movement restrictions on selected agricultural businesses, cargo transportation, and the sale of farming supplies were ultimately relaxed in regions where the virus's spread was deemed to be contained (Ray & Subramanian, 2020).

**Fig. 2:**
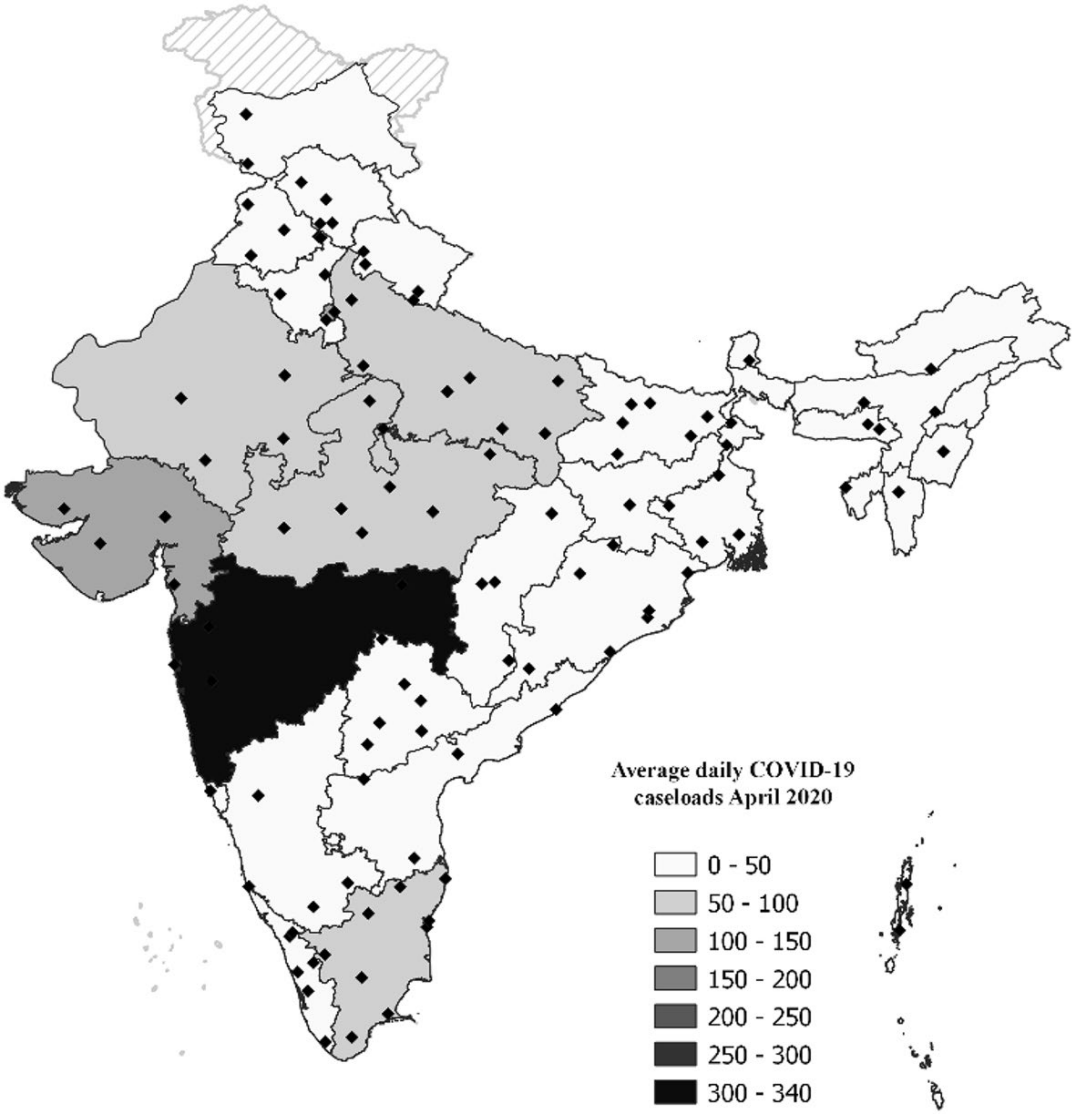
State-wise variation in COVID-19 caseloads. Source: Created by authors. COVID-19 data from the COVID-19 India tracker. Data accessed 13.05.2021. The dots in the map represent the locations of the sample of markets in our study

Since our econometrics analysis uses data up to June 2020, we present in Fig. 1 the incidence of COVID-19 caseloads up to that date. The dotted line shows the date of the first national lockdown. Figure 1 shows that India imposed its national lockdown at a time when COVID-19 infection was relatively low. Figure 2 presents the incidence of COVID-19 at the state level in India for April 2020. As can be seen, there was substantial variation in COVID-19 infections in the initial months of the pandemic. Maharashtra had the largest daily confirmed COVID-19 cases, followed by Gujarat and New Delhi. As a result, state-level administrations' reactions to COVID-19 have been mixed, with some states, like Punjab and Telangana, prolonging the lockdown till June 30th, and others starting their lockdown several days before the national lockdown (Lowe et al., 2021). We use this variation in the spread of COVID-19 caseloads to study the effects on urban food markets in India.

### Potential channels of impacts of the spread of COVID-19 on urban markets

**Fig. 3:**
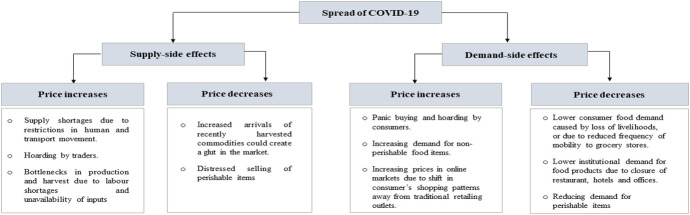
Channels of impacts of the spread of COVID-19 on urban markets. Source: created by authors

The spread of COVID-19 and the related measures taken to curb the contagion of the virus could affect short-term market outcomes due to both supply and demand-side issues (Fig. 3). On the supply side, food prices in wholesale and retail markets could increase due to a severe shortage of commodities, especially in deficit markets caused by restrictions on human and transport movements. It could also increase due to artificial shortages created by traders due to hoarding, especially for storable food items which have a good post-harvest infrastructure. Moreover, prices could increase because of bottlenecks in production and harvest due to labor shortages and unavailability of inputs across the value chain. It is expected that because of these disruptions, the quantities of commodities arriving at urban wholesale markets—which also supply downstream retail outlets—will decrease or slow down, putting upward pressure on wholesale prices (Mahajan & Tomar, 2021; Varshney et al. 2020). The extent to which price increases at the wholesale level are passed on to the retail level will depend on the power relations between wholesale and retail traders. On the other hand, additional transaction costs caused by the containment measure could have differential effects at the wholesale and retail levels, i.e., market closures affect retail traders much stronger than wholesale traders.

While in general, we expect a supply shock such as the one COVID-19 imposed to increase overall prices, prices could also fall for some perishable items such as vegetables which were harvested around the time of the onset of COVID-19 and have limited cold storage facilities (Bairagi et al., 2022). The increased arrivals of the commodities and limited consumer demand could create a glut in the market and cause ‘distressed sales’ which could severely bring down prices and have severe negative consequences on the incomes of smallholder farmers. Therefore, the overall effect on prices would depend on the type of commodity—perishable or storable. Further, on the demand side, the spread of COVID-19 could affect urban food markets in several ways which could either have a positive or a negative effect on prices. On one hand, prices could fall due to lower consumer food demand caused by loss of livelihoods, or due to reduced frequency of mobility to grocery stores, or low shelf-life of commodities such as vegetables. It could also fall due to lower institutional demand for food products due to the closure of restaurants, hotels, and offices. On the other hand, prices could increase for some items due to higher food demand because of panic buying and hoarding by consumers (Akter, 2020). Price effects can also be observed if the COVID-19 changes the composition of the consumer food basket by increasing demand for non-perishable storable food items and reducing demand for perishable items. Moreover, it could alter consumers’ shopping patterns by shifting demand from traditional retailing outlets such as wet markets, supermarkets, and local stores towards online formats of shopping, thereby increasing prices in online markets and reducing prices in brick and motor shops (Reardon et al., 2021).

In addition to the overall price effects in retail and wholesale markets caused by COVID-19-related restrictions, we also expect that disruptions in upstream food supply chains will affect retail markets via wholesale market disruptions. In India, disruptions in the wholesale markets are less likely to affect disruptions in the retail markets for cereals compared to non-perishables and perishables traded in the open market. This is because the prices of cereals such as rice and wheat are controlled by the Government of India through its Minimum Support Price (MSP) program. Therefore, it is likely that markets for these commodities witnessed fewer distortions in mark-up between wholesale and retail prices. Non-perishable commodities traded in the open market are likely to have experienced larger distortions; to what extent depends on power relations between farmers, wholesale and retail traders, and middlemen. In contrast, price distortions for perishable items such as vegetables could have declined despite a drop in wholesale market arrivals. This is because of the short shelf-life of these commodities as well as weak consumer demand which could limit traders’ ability to increase prices (Rawal and Verma 2020). Finally, we also expect those constraints in transportation and human movement to increase transaction costs across all value chains, thus resulting in greater spatial price dispersion between markets in both retail and wholesale markets for all commodities.

## Materials and methods

### Data

We construct a market-level monthly balanced panel dataset, recording retail and wholesale prices of 15 commodities^4^ across markets spreading over 33 states and union territories (UTs) of India for the period January 2019–June 2020. We construct a sample of 2052 observations including 114 cross-sectional units spanning over 18 months. Figure 2 plots the location of all the markets in our sample on the Indian map. We construct this dataset from several publicly available secondary sources of data. Daily market-wise data on retail and wholesale prices of different commodities were collected from the website of the Government of India’s Department of Consumer Affairs.^5^ All price data are deflated by the wholesale price index at 2012 prices given by the Office of the Economic Adviser.^6^ To capture the incidence of COVID-19 at the state level, we use publicly available data from the COVID-19 India tracker,^7^ which is a crowd-sourced database for real-time COVID-19 statistics at the state level. It curates data from Government sources such as the Ministry of Health and Family Welfare and data published through bulletins and press releases by the state health department from all states and UTs in India. Although our price data are at the market level, we use state-level data on COVID-19 incidence in our main econometric specifications for two reasons. First, at the onset of the pandemic, due to limited testing capacity and unavailability of personal protective equipment (PPE) in smaller towns and peri-urban regions, there is a possibility that daily COVID data at the district level is likely to be underreported. It is also likely that the difference in under-reporting varies systematically across districts that have poor infrastructure and are remotely located. Second, the retail and price data collected from the Department of Consumer Affairs consists of data from 114 markets, while the corresponding district-level COVID-19 data are available only for a sub-set of 77 markets. We, however, use the district-level data on COVID-19 as a robustness check in the Online Appendix.

Furthermore, because demand and supply-side factors, as well as climatic conditions, influence food prices, we incorporate many time-varying variables that account for these aspects in our econometric analysis. Monthly rainfall data were extracted from satellite-based precipitation datasets provided by the Center for Hydrometeorology and Remote Sensing (CHRS).^8^ We use CHRS’s PERSIAN-Cloud Classification System (CCS) dataset which estimates global rainfall in near real-time and at high spatial resolution (0.04° × 0.04° or 4 km × 4 km). Data from CHRS is available in raster format, which is then converted to numerical values using Geographic Information Systems (GIS) programs. Since the rainfall data is available at 0.04° × 0.04° spatial resolution, we average the rainfall for each state-level political boundary for each month to get the average month-wise rainfall data at the state level. We use rainfall at the state level because most markets source produces from production hubs within a state and not necessarily just around these markets. Therefore, rainfall data at the aggregate level is a better proxy for climatic conditions within a region. Further, daily diesel prices were collected from the Petroleum Planning and Analysis Cell.^9^ To control for demand-side effects such as consumer demand, we include nightlight radiance, which is often used as an indicator for economic output and growth at the sub-national level (Gibson et al., 2021; Mellander et al., 2015; Roberts, 2021). We obtain the data from the World Bank Light Every Night data repository, which provides imaginary data produced by the Visible Infrared Imaging Radiometer Suite (VIIRS) from the Suomi National Polar Partnership (SNPP) satellite. The VIIRS provides, as part of its Day–Night Band (DNB) instrument, low light imaging data at night. The light intensification of the VIIRS DNB makes artificial light detectable. To generate index data for each market, we generate a single image based on all 742 m × 742 m pixels within a radius of 50 km around the geographical location of the market. We compute the monthly nightlight index as the average of the DNB radiance values of that image during the respective month. However, daily and monthly radiance is not free of ephemeral lights and background radiance (Gibson et al., 2021), some of which are caused by seasonal variation in airflow. Therefore, to obtain nightlight radiance free of potential correlation between seasonality and economic activity, we correct the monthly index by the average index over the past 3 years in the same months.

### Methodology

This paper analyses the links between the ongoing pandemic and market performance in urban areas in India up to June 2020. Specifically, we examine how the gradual spread of the COVID-19 pandemic affected (1) price levels, (2) vertical price difference between retail and wholesale prices, and (3) spatial price dispersion between markets.

The analysis requires a quasi-experimental design given that the whole of India was, albeit at different times and to a different extent, exposed to COVID-19 induced shocks. Yet, the proper identification of the COVID-19 impact requires a comparison between treated and untreated markets. One option is to consider the implementation of the COVID-19 contamination measures as the treatment, however, in this case, the nationwide lockdown at the end of March 2020 would make all markets treated cases without establishing untreated counterfactuals. In addition to that, food trade logistics were exempted from movement restriction, and therefore, the national lockdown was not the singular reason for value chains disruptions. Instead, we follow Varshney et al. (2020) who defined a treated market as a market located in a region where COVID-19 increased faster, namely we use 100 confirmed caseloads as a cut-off at the respective state.^10^ The rationale is that: as regions crossed a certain threshold of COVID-19 cases the impact on market outcomes would be larger because of stricter vigilance by authorities in the adherence to the restrictions, or due to the closure of agriculture market yards. Additionally, people themselves engaged in voluntary precautionary measures which could have resulted in larger distortions in consumer demand and supply-side bottlenecks. Thus, in this study, the event is defined as the time *t* in which market *i* located in state *s* crosses the confirmed caseloads cut-off. We admit that confirmed caseloads understate the true incidence of COVID-19 contagion due to insufficient testing and under-reporting. However, confirmed cases are an important indicator for policy decisions and so can provide insight into the relationship between the spread of COVID-19 and market outcomes. (Lowe et al., 2021). Table 1 gives a summary of the timing of the event occurring between March and June 2020 and Table A2 in the Appendix gives more detailed information about the timings of each region crossing the caseload threshold. We also use three alternative definitions for the spread of COVID-19 and related shocks to markets as robustness checks to our main specification, namely: (1) we re-estimate our results by moving the cut-off from 100 to 300, (2) we re-define our treatment variable using a continuous variable that measures average daily confirmed COVID-19 caseloads, and (3) we use a dummy variable to capture the month when the national lockdown was implemented. We discuss this further in Sect. 3.2.2.

**Table 1:** Timing of event between March and June 2020

	Number of markets	
	Event occurred	Event yet to occur
March 2020	1	113
April 2020	10	104
May 2020	51	63
June 2020	89	25

Event is defined as state s crosses the 100 caseloads cut-off

#### Spread of COVID-19 and urban markets

*Association between the spread of COVID-19 and prices levels* To analyze the association between the spread of COVID-19 and market prices, we use a fixed-effect panel regression model such as:

1 $$Ln(p)_{ist} = \alpha {\text{COVID}}19_{ist} + X^{\prime}_{ist} b + M^{\prime}_{t} c + d{\text{Trend}}_{it} + \mu_{i} + \lambda_{t} + \varepsilon_{ist} .$$

The outcome variables of interest in Eq. (1) are the natural logarithm of retail, wholesale prices, or mark-up between retail and wholesale prices^11^ in market *i*, located in state *s*, and in time *t*. ${\text{COVID}}19_{ist}$ is a dummy variable that takes a value of 1 for all months after which a market *i* located in state *s* crosses the COVID-19 confirmed caseload threshold. As a robustness check, we also re-estimate Eq. (1) using COVID-19 caseloads at the district level. We limit our data till June 2020, such that some markets work as pure controls as they crossed the cut-off later (see Table 1 and Table A2 in the Online Appendix for more details). Further, in our specifications, we also include several time-varying factors ($X_{st}$) that control for supply and demand-side factors and climatic conditions. To capture supply-side factors, we use diesel prices to control transportation costs; demand-side factors are captured through a night light index to control economic activity around a market. Since studies have shown that the COVID-19 crisis-affected overall intensity of light (Roberts, 2021), we also show these results excluding the night light variables as a robustness check. Further, climatic conditions are captured by average rainfall in the state where the market is located. $M_{t}$ is a vector of month dummies to control for seasonality in markets. Additionally, we also expect that markets located in different geographical regions of India to have different seasonal patterns; therefore, we interact the month dummies with region dummies. Further, most time-series data are usually subject to a trend component, therefore we include time trends in our specification. $\mu_{i}$ are market fixed effects to control for all unobserved time in-variant differences between markets such as geographical location, market size, and population density. $\lambda_{t}$ are year-fixed effects to control for systematic differences between observed time units. $\varepsilon_{ist}$ is a random error term with zero conditional means. We cluster standard errors by markets to account for spatial correlation in our main specifications. As a robustness check, we also show the results with standard errors clusters by time to account for time dependencies. $\alpha$ is our primary coefficient of interest and it captures the market effects of the spread of COVID-19.

*Association between the spread of COVID-19 and price dispersion* To estimate the association between the spread of COVID-19 and price dispersion between major markets in India, we create a dyadic market dataset. We estimate a fixed-effects panel regression model such as the following specification:

2 $$\left| {p_{it} - p_{jt} } \right| = \gamma {\text{COVID}}19_{ij,t} + X_{ij,t}^{^{\prime}} b + M_{t}^{^{\prime}} c + d{\text{Trend}}_{ij,t} + \theta_{ij} + \lambda_{t} + \varepsilon_{ij,t} ,$$

where $\left| {p_{it} - p_{jt} } \right|$ is the log of absolute price dispersion of prices in the market *i* and *j* at time *t.* ${\text{COVID}}19_{ij,t}$ is a dummy variable equal to 1 if either market *i* or market *j* crosses the 100-caseload threshold in time *t*, otherwise 0*.* $X_{ij,t}$ is a vector of time-variant contextual variables that affect price dispersion between two markets. $\theta_{ij}$ are market-pair fixed effects to control for all unobserved time in-variant difference between market-pairs and $\lambda_{t}$ are time-fixed effects. $\varepsilon_{ij,t}$ is a random error term. Like the earlier specification, we also include month dummies to control for seasonality and a trend variable to capture the time-series trend component. Here, we are interested in the point estimate *γ*. Typically, when there are spatial price differences between two markets, market agents usually take advantage of arbitrage opportunities and move produce to markets offering higher prices. When markets are functioning efficiently, the spatial price dispersion should decline, while high transaction costs and disruptions in markets should increase the price dispersion between markets. Therefore, with the spread of COVID-19 and related supply bottlenecks, we expect *γ* to be positive for most commodities.

#### Robustness checks

We conduct several robustness checks of our estimates. First, since the time-variant control variables such as night light and diesel prices are likely to be affected by COVID-19, we also re-estimate Eqs. (1) and (2) by removing these two variables as a robustness check. Second, cross-sectional time-series data are often fraught with issues of time dependencies in the stochastic error term. Therefore, to account for this issue we re-estimate both the equations by clustering standard errors by quarters of the year. Third, similar to Varshney et al. (2020) we re-estimate Eq. (1) by moving the cut-off for the treatment variable from 100 to 300. Fourth, we re-define our treatment variable using a continuous variable. Here, we use the inverse hyperbolic sine transformation of the average daily COVID-19 caseloads in month *t* as an indicator of the intensity of contamination in the market *i* located in the state *s*. This allows us to retain zero values during pre-COVID periods. The specification is identical to Eqs. (1) and (2), except now the ${\text{COVID}}19_{ij,t}$ variable is continuous. Thus, the coefficient in this alternative specification is interpreted as for every 1 percentage increase in COVID-19 caseloads in a given market, prices increase or decrease by $\alpha$ percentage, and price dispersion between markets *i* and *j* increase or decrease by $\gamma_{1}$ percentage. Finally, we also estimate another alternative specification by defining our treatment variable as a dummy variable that captures the month when the national lockdown was implemented to understand the effects of the COVID-19-induced supply shocks where COVID-19 spread first. We, therefore, estimate the following specification with retail and wholesale prices as our outcome variable:

3 $$Ln(p)_{ist} = \alpha_{1 } {\text{After}}_{t} + \alpha_{2 } {\text{COVID}}19_{ist} + \alpha_{3} {\text{After}}_{t} \times {\text{COVID}}19_{ist} + X_{ist}^{^{\prime}} b + M_{t}^{^{\prime}} c + d{\text{Trend}}_{it} + \mu_{i} + \delta_{t} + \varepsilon_{ist} .$$

Here, the specifications are similar to Eq. (1), except we have two additional terms. First, a dummy variable ${\text{After}}_{t}$, which takes a value of 1 for all markets after the implementation of India’s national lockdown. Second, we add an interaction term between ${\text{COVID}}19$ and ${\text{After}}_{t}$. In Eq. (3), we are mainly interested in the coefficient $\alpha_{3}$. The coefficient of the interaction term estimates the differential effects of national lockdown in markets where COVID-19 caseloads crosses the threshold.

## Results and discussion

### Descriptive statistics

**Fig. 4:**
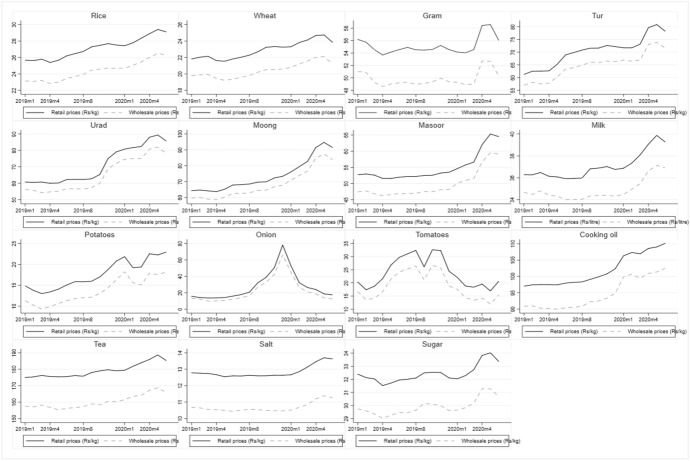
Average retail and wholesale prices of food items (January 2019–June 2020). All price data are deflated by the wholesale price index at 2012 prices. Pulses: Gram (Chickpea), Tur (Pigeon pea), Urad (Black gram), Moong (Yellow lentils), Masoor (Red lentils)

We present the descriptive statistics of all our outcome and control variables disaggregated by the period before and after the event in Table A3 in the Online Appendix. As can be seen, without controlling for other factors, the student t-statistics results suggest that the average retail and wholesale prices for most non-perishable commodities increased after the event,^12^ while prices of perishables such as onions and tomatoes declined. We also observe a significant increase in the mark-ups between retail and wholesale prices of pulses and potatoes after the event. Figure 4 presents commodity-wise trends in average retail and wholesale prices for all of India for the period January 2019–June 2020. Here, we see that prices of all commodities in retail and wholesale markets were gradually increasing since the beginning of 2019; however, for onions and tomatoes, we see a downward trend in prices since the end of 2019.

### Association between the spread of COVID-19 and market outcomes

#### Retail and wholesale prices

To analyze the link between COVID-19 and prices in urban markets, we present in Table 2 the summary results of Eq. (1) with retail prices as the outcome variable and in Table 3 we present the results for wholesale prices. We are interested in the coefficient on the dummy variable ${\text{COVID}}19_{ist}$. All regressions are with year-fixed effects, market fixed effects, time trends, monthly time dummies, and state-month fixed effects. In addition to that, we include time-varying controls such as night light intensity, diesel prices, and rainfall. Here, we classify commodities as storable and perishable items. Rice, wheat, gram (chickpea), tur (pigeon pea), urad (black chickpea), moong (yellow lentils), masoor (red lentils), packaged oil, tea, salt, and sugar are storable items, while milk, onion, potatoes, and tomatoes are perishable items. Tables 2 and 3 suggest that the spread of COVID-19 had a statistically significant effect on retail and wholesale prices for most commodities, but the sign of the effect varies depending on the type of commodity. Prices of rice and wheat increased by roughly 3% and 3–4%, respectively, whereas pulses increased by about 3–8%. Furthermore, milk prices increased by 2–3%, while prices of processed foods such as packaged oils, salt, and sugar increased by 2–4%. In the case of vegetables, we find that prices of perishable commodities like tomatoes dropped by as much as 17–22%, while onions fell by 42–48%. As a robustness check, we also show the results with the district-level COVID-19 caseloads in Table A6 and Table A7 in the Online Appendix. The specification is like Eq. (1), except the dummy variable COVID-19 takes a value of 1 for all months after which a market located in a particular district crosses the COVID-19 confirmed caseload threshold. It is reassuring that for most commodities the results are similar in significance and magnitude to the ones presented in Tables 2 and 3. In general, the results suggest that prices increased for commodities with longer shelf-life, while perishable commodities such as onions and tomatoes witnessed a decline in prices.

**Table 2:** Spread of COVID-19 and retail prices (summary results)

Panel A	(1)	(2)	(3)	(4)	(5)
	Rice	Wheat	Gram	Tur	Urad
COVID 19^a^ (dummy)	0.026** (0.012)	0.040*** (0.011)	0.043*** (0.013)	0.032** (0.015)	0.059** (0.023)
Controls included	Yes	Yes	Yes	Yes	Yes
Year fixed effects	Yes	Yes	Yes	Yes	Yes
Market fixed effects	Yes	Yes	Yes	Yes	Yes
Group-specific time trend	Yes	Yes	Yes	Yes	Yes
Monthly time dummies	Yes	Yes	Yes	Yes	Yes
State × monthly time dummies	Yes	Yes	Yes	Yes	Yes
Observations	1971	1824	1961	1946	1966
R-squared	0.694	0.596	0.451	0.753	0.842

Panel B	(6)	(7)	(8)	(9)	(10)
	Moong	Masoor	Milk	Onion	Potatoes
COVID 19^a^ (dummy)	0.054*** (0.016)	0.083*** (0.014)	0.022*** (0.007)	− 0.416*** (0.049)	0.108*** (0.026)
Controls included	Yes	Yes	Yes	Yes	Yes
Year fixed effects	Yes	Yes	Yes	Yes	Yes
Market fixed effects	Yes	Yes	Yes	Yes	Yes
Group-specific time trend	Yes	Yes	Yes	Yes	Yes
Monthly time dummies	Yes	Yes	Yes	Yes	Yes
State × monthly time dummies	Yes	Yes	Yes	Yes	Yes
Observations	1971	1966	1964	1971	1971
R-squared	0.841	0.701	0.755	0.899	0.793

Panel C	(11)	(12)	(13)	(14)	(15)
	Tomatoes	Packaged oils	Tea	Salt	Sugar
COVID 19^a^ (dummy)	− 0.172*** (0.050)	0.019** (0.008)	0.019 (0.015)	0.040*** (0.014)	0.030*** (0.007)
Controls included	Yes	Yes	Yes	Yes	Yes
Year fixed effects	Yes	Yes	Yes	Yes	Yes
Market fixed effects	Yes	Yes	Yes	Yes	Yes
Group-specific time trend	Yes	Yes	Yes	Yes	Yes
Monthly time dummies	Yes	Yes	Yes	Yes	Yes
State × monthly time dummies	Yes	Yes	Yes	Yes	Yes
Observations	1967	1971	1956	1971	1971
R-squared	0.658	0.772	0.504	0.552	0.469

Regressions are conducted for the period Jan 2019–June 2020

*Significant at 10% level, **significant at 5% level, ***significant at 1% level.

^a^Daily COVID 19 caseloads cross 100 for a specific state in which market *i* is located. Nominal prices series have been deflated by the wholesale price index (2011–12 prices) and then all prices are log-transformed. Standard errors are clustered by markets in parenthesis. Pulses: Gram (Chickpea), Tur (Pigeon pea), Urad (Black gram), Moong (Yellow lentils), Masoor (Red lentils). The full regression models are presented in Table A4 in the Online Appendix. Regression results with district-level caseloads are presented in Table A6

**Table 3:** Spread of COVID-19 and wholesale prices (summary results)

Panel A	(1)	(2)	(3)	(4)	(5)
	Rice	Wheat	Gram	Tur	Urad
COVID 19^a^ (dummy)	0.033*** (0.010)	0.049*** (0.010)	0.031*** (0.011)	0.020 (0.014)	0.052** (0.020)
Controls included	Yes	Yes	Yes	Yes	Yes
Year fixed effects	Yes	Yes	Yes	Yes	Yes
Market fixed effects	Yes	Yes	Yes	Yes	Yes
Group-specific time trend	Yes	Yes	Yes	Yes	Yes
Monthly time dummies	Yes	Yes	Yes	Yes	Yes
State × monthly time dummies	Yes	Yes	Yes	Yes	Yes
Observations	1954	1797	1945	1926	1947
R-squared	0.706	0.595	0.460	0.737	0.849

Panel B	(6)	(7)	(8)	(9)	(10)
	Moong	Masoor	Milk	Onion	Potatoes
COVID 19^a^ (dummy)	0.047*** (0.014)	0.072*** (0.011)	0.031*** (0.009)	− 0.478*** (0.055)	0.077*** (0.028)
Controls included	Yes	Yes	Yes	Yes	Yes
Year fixed effects	Yes	Yes	Yes	Yes	Yes
Market fixed effects	Yes	Yes	Yes	Yes	Yes
Group-specific time trend	Yes	Yes	Yes	Yes	Yes
Monthly time dummies	Yes	Yes	Yes	Yes	Yes
State × monthly time dummies	Yes	Yes	Yes	Yes	Yes
Observations	1954	1940	1434	1954	1953
R-squared	0.842	0.744	0.755	0.897	0.822

	(11)	(12)	(13)	(14)	(15)
	Tomatoes	Packaged oils	Tea	Salt	Sugar
COVID 19^a^ (dummy)	− 0.223*** (0.054)	0.017** (0.008)	0.021*** (0.008)	0.026* (0.014)	0.025*** (0.006)
Controls included	Yes	Yes	Yes	Yes	Yes
Year fixed effects	Yes	Yes	Yes	Yes	Yes
Market fixed effects	Yes	Yes	Yes	Yes	Yes
Group-specific time trend	Yes	Yes	Yes	Yes	Yes
Monthly time dummies	Yes	Yes	Yes	Yes	Yes
State × monthly time dummies	Yes	Yes	Yes	Yes	Yes
Observations	1943	1947	1667	1910	1954
R-squared	0.670	0.774	0.606	0.615	0.506

Regressions are conducted for the period Jan 2019–June 2020

*Significant at 10% level, **significant at 5% level, ***significant at 1% level

^a^Daily COVID 19 caseloads cross 100 for a specific state in which market i is located. Nominal prices series have been deflated by the wholesale price index (2011–12 prices) and then all prices are log-transformed. Standard errors are clustered by markets in parenthesis. Pulses: Gram (Chickpea), Tur (Pigeon pea), Urad (Black gram), Moong (Yellow lentils), Masoor (Red lentils). The full regression models are presented in Table A5 in the Online Appendix. Regression results with district-level caseloads are presented in Table A7

In general, the findings for wholesale and retail prices across different commodities are very similar. However, it is important to note that retail prices are always higher than wholesale prices in absolute terms, sometimes up to 20% higher. Therefore, the COVID-19-related price increase in absolute terms is stronger for retail than for wholesale prices. This indicates that transaction costs are passed on to the consumer. In addition to that, it suggests that containment measures, such as market closures, may have caused higher disruptions at the retail than at the wholesale level.

Cereals and pulses are important food items in Indian diets, while packaged oils, salt, and sugar are complimentary food items in Indian cooking. Prices of non-perishable items likely increased because of higher demand for essential commodities with longer shelf-life due to panic buying and stockpiling by consumers as well as supply-side market disruptions in the movement of stocks from production hubs to wholesale and retail markets. Packaged milk, while it is a perishable item, the COVID-19 pandemic increased the demand for ultra-high temperature (UHT) treated milk in tetra-packs which has a shelf-life of about 3–6 months.^13^ Increased demand for packaged milk and supply distortions especially in milk deficit regions increased the prices of milk. On the other hand, perishable items such as vegetables saw a decline in prices probably due to an oversupply in production centers. For example, the implementation of COVID-19-related restrictions coincided with the harvest of ‘late Kharif’ and ‘Rabi’ season crops (Government of India, 2020a, 2020c), which could have caused a glut in production centers. In India, the ‘late Kharif” onion crop is transplanted around October–November, and it is harvested around January–March, while transplanting of the ‘Rabi’ crop is done in December–January and it is harvested around the end of March–May. These two seasons account for about 85% of India’s total onion production (Government of India, 2020a). Further, the closure of India’s largest onion market yard (Lasalgoan) due to social distancing protocols implemented by the local administration, and low institutional demand resulted in distressed sales thus affecting onion prices significantly.^14^ Similarly, for tomatoes, the months of December to June are important for the sale of tomatoes since the ‘Rabi’ crop is ready for harvest. ‘Rabi’ harvest accounts for about 75% of India’s total annual tomato production (Government of India, 2020c). In general prices of these two vegetables are lower in March–May due to the harvest of the crop. However, our results suggest that even after controlling for seasonality in production, prices declined significantly due to COVID-19 at the onset of the pandemic.^15^ We think that higher production, low demand, and market disruptions such as traders not coming to market yards, closure of market yards, inter-state movement obstacles, and labor shortages might have resulted in an oversupply for both these vegetables and brought down prices. Varshney et al. (2020) and Rawal and Verma (2020) also find suggestive evidence that prices of onions and tomatoes were trending downwards. Rawal and Verma (2020) also show using descriptive statistics that arrivals of these two crops in agricultural markets declined by 70% and 26%, respectively, compared to the previous year. They argue that, despite a drop in market availability, traders were unwilling to charge a high price due to interruptions in the downstream supply chain and low demand caused by lost income and jobs. Our findings are also consistent with the findings of Bairagi et al. (2022) and Ceballos et al. (2020) who find that prices of onions and tomatoes declined due to the nationwide lockdown. COVID-19-related restrictions, in general, resulted in a severe labor scarcity during harvest season, limiting or delaying harvest and post-harvest activities, as well as the capacity to mobilize labor to transport produce to markets. In addition, travel restrictions limited traders from procuring produce at the farmgate (Ceballos et al., 2020). In the case of perishable goods, such increased transaction costs would result in a large amount of unsold inventory, distressed sales, and significant losses for farmers.

Unlike onions and tomatoes, prices for potatoes increased by around 8–11%, even when about 67% of total potato production is harvested around December–March (Government of India, 2020b). This is probably because potatoes have a relatively higher shelf-life. It is also the third most well-stocked agriculture commodity in India after rice and wheat because of the existence of cold storage operated by the private sector (Tata-Cornell Institute, 2020). So, prices of potatoes could have increased due to increased demand, stockpiling by traders, and disturbance in the movement of produce.

These results indicate severe consequences for consumers, especially those living below the poverty line as well as smallholder farmers producing perishable items. As the cost of staple food rises, food accessibility is significantly reduced. Further, a significant drop in perishables prices owing to distressed sales might have serious consequences for vegetable growing smallholder farmers' income and food security.

#### Vertical and spatial price dispersions

In Table 4 we show the summary results of the association between the spread of COVID-19 and vertical spread in retail and wholesale prices. These estimates help us understand how disruptions in upstream food supply chains are likely to affect the mark-ups between retail and wholesale prices. We find that for items with longer shelf-life such as pulses, and potatoes, the absolute price gap between the retail and wholesale prices increased. The highest mark-ups were observed for pulses (13–19%), and potatoes (13%). We do not find a significant effect of COVID-19 on mark-ups between retail and wholesale markets for cereals (rice and wheat), which are controlled by the Government’s minimum support prices. This is in line with findings by Nasir and Mulyo (2021) who analyze market integration in Indonesia’s rice sector—which is also controlled by the government—during the COVID-19 pandemic. Further, perishable items such as onions, and tomatoes, saw a decrease in the absolute price difference between the retail and wholesale markets, however, the effect is not statistically significant for tomatoes. Moreover, in tandem with the overall decline in retail and wholesale prices of onions, overall mark-up also came down by about 31%. This hints that, despite a drop in wholesale market arrivals, traders were unable to raise prices of short-shelf-life produce due to interruptions in downstream supply chains, inability to store them for the future, and weak consumer demand. We show the results with COVID-19 caseloads at the district level in Table A9 in the Online Appendix. These results are similar in terms of the direction of the price change but differ in the magnitude of the effect. For example, using the district-level caseloads, we find that the absolute price difference between retail and wholesale prices increased by 14–15% for pulses, while potato prices increased by 20%, and onion prices declined by 21%.

**Table 4:** Spread of COVID-19 and vertical spread (retail-wholesale) (summary results)

	(1)	(2)	(3)	(4)	(5)
	Rice	Wheat	Gram	Tur	Urad
COVID-19^a^ (dummy)	0.002 (0.045)	0.009 (0.043)	0.127** (0.050)	0.186*** (0.063)	0.116 (0.080)
Controls included	Yes	Yes	Yes	Yes	Yes
Year fixed effects	Yes	Yes	Yes	Yes	Yes
Market fixed effects	Yes	Yes	Yes	Yes	Yes
Group-specific time trend	Yes	Yes	Yes	Yes	Yes
Monthly time dummies	Yes	Yes	Yes	Yes	Yes
State × monthly time dummies	Yes	Yes	Yes	Yes	Yes
Observations	1,954	1,796	1,944	1,926	1,946
R-squared	0.401	0.475	0.530	0.545	0.499

	(6)	(7)	(8)	(9)	(10)
	Moong	Masoor	Milk	Onion	Potatoes
COVID-19^a^ (dummy)	0.150** (0.068)	0.188*** (0.066)	− 0.047 (0.056)	− 0.310*** (0.063)	0.134** (0.058)
Controls included	Yes	Yes	Yes	Yes	Yes
Year fixed effects	Yes	Yes	Yes	Yes	Yes
Market fixed effects	Yes	Yes	Yes	Yes	Yes
Group-specific time trend	Yes	Yes	Yes	Yes	Yes
Monthly time dummies	Yes	Yes	Yes	Yes	Yes
State × monthly time dummies	Yes	Yes	Yes	Yes	Yes
Observations	1,954	1,937	1,432	1,954	1,953
R-squared	0.525	0.421	0.461	0.659	0.461

	(11)	(12)	(13)	(14)	(15)
	Tomatoes	Packaged oils	Tea	Salt	Sugar
COVID-19^a^ (dummy)	− 0.059 (0.067)	0.089** (0.045)	0.211*** (0.067)	0.064** (0.030)	0.058 (0.046)
Controls included	Yes	Yes	Yes	Yes	Yes
Year fixed effects	Yes	Yes	Yes	Yes	Yes
Market fixed effects	Yes	Yes	Yes	Yes	Yes
Group-specific time trend	Yes	Yes	Yes	Yes	Yes
Monthly time dummies	Yes	Yes	Yes	Yes	Yes
State × monthly time dummies	Yes	Yes	Yes	Yes	Yes
Observations	1,943	1,947	1,666	1,910	1,954
R-squared	0.447	0.425	0.553	0.533	0.431

Regressions are conducted for the period Jan 2019–June 2020

*Significant at 10% level, **significant at 5% level, ***significant at 1% level

^a^Daily COVID 19 caseloads cross 100 for a specific state in which market i is located.Nominal prices series have been deflated by the wholesale price index (2011–12 prices). The dependent variable is measured as a log of the absolute price difference between retail and wholesale prices. Standard errors are clustered by markets in parenthesis. Pulses: Gram (Chickpea), Tur (Pigeon pea), Urad (Black gram), Moong (Yellow lentils), Masoor (Red lentils). The full regression models are presented in Table A8 in the Online Appendix. Regression results with district-level caseloads are presented in Table A9

In Tables 5 and 6, we present the results of Eq. (2), wherein we estimate the association between the spread of COVID-19 and spatial price dispersion between retail and wholesale markets in India, respectively. In the retail segment, as COVID-19 infection spread across markets, we see a significant increase in price dispersion for most commodities. The largest market distortion was observed for pulses and tomatoes. For example, retail price dispersion increased by 30–56% for pulses and 35% for tomatoes. The results also suggest that retail price dispersion increased for wheat, potatoes, and packaged oils, while price dispersion declined for onions. In the wholesale market too, we see significant market distortions but the magnitude for some commodities is less than in the retail market (Table 7). For example, wholesale price dispersion in the Gram (chickpea) market increased by 24% compared to 56% in the retail market, and wholesale tomatoes prices increased by 17% compared to 35% in retail prices. This is also true for yellow lentils and potatoes. These results suggest that with the spread of COVID-19, the measures undertaken to reduce contagion had a significant effect on price dispersion between markets as well as mark-ups between retail and wholesale food markets.

**Table 5:** Spread of COVID-19 and spatial retail spread (market j and k) (summary results)

	(1)	(2)	(3)	(4)	(5)
	Rice	Wheat	Gram	Tur	Urad
COVID-19^a^ (dummy)	0.055 (0.044)	0.165*** (0.053)	0.562*** (0.052)	0.298*** (0.068)	0.046 (0.047)
Controls included	Yes	Yes	Yes	Yes	Yes
Monthly time dummies	Yes	Yes	Yes	Yes	Yes
Group-specific trend	Yes	Yes	No	No	Yes
Common time trend	No	No	Yes	Yes	No
Quadratic time trend		No	Yes	Yes	No
Year fixed effects	Yes	Yes	Yes	Yes	Yes
Market pair fixed effects	Yes	Yes	Yes	Yes	Yes
Observations	10,868	7842	10,547	10,200	10,867
R-squared	0.325	0.375	0.032	0.035	0.345

	(6)	(7)	(8)	(9)	(10)
	Moong	Masoor	Milk	Onion	Potatoes
COVID-19^a^ (dummy)	0.343*** (0.060)	0.300*** (0.055)	0.016 (0.032)	− 0.151** (0.065)	0.146*** (0.053)
Controls included	Yes	Yes	Yes	Yes	Yes
Monthly time dummies	Yes	Yes	Yes	Yes	Yes
Group-specific trend	Yes	Yes	No	Yes	Yes
Common time trend	No	No	Yes	No	No
Quadratic time trend	No	No	Yes	No	No
Year fixed effects	Yes	Yes	Yes	Yes	Yes
Market pair fixed effects	Yes	Yes	Yes	Yes	Yes
Observations	11,046	11,001	10,655	11,059	11,083
R-squared	0.238	0.246	0.025	0.129	0.143

	(11)	(12)	(13)	(14)	(15)
	Tomatoes	Packaged oils	Tea	Salt	Sugar
COVID-19^a^ (dummy)	0.351*** (0.055)	0.097** (0.047)	0.027 (0.030)	0.027 (0.035)	0.027 (0.045)
Controls included	Yes	Yes	Yes	Yes	Yes
Monthly time dummies	Yes	Yes	Yes	Yes	Yes
Group-specific trend	No	Yes	No	Yes	Yes
Common time trend	Yes	No	Yes	No	No
Quadratic time trend	Yes	No	Yes	No	No
Year fixed effects	Yes	Yes	Yes	Yes	Yes
Market pair fixed effects	Yes	Yes	Yes	Yes	Yes
Observations	11,031	11,157	10,503	10,211	10,827
R-squared	0.044	0.300	0.004	0.289	0.212

Regressions are conducted for the period Jan 2019–June 2020. The dependent variable is the log-transformed absolute price difference in retail prices between market *j* and market *k*

*Significant at 10% level, **significant at 5% level, ***significant at 1% level

^a^COVID-19 takes a value of 1 if at least one market crosses the 100-caseload threshold. Nominal prices series have been deflated by the wholesale price index (2011–12 prices) and then all prices are log-transformed. Pulses: Gram (Chickpea), Tur (Pigeon pea), Urad (Black gram), Moong (Yellow lentils), Masoor (Red lentils). The full regression models are presented in Table A10 in the Online Appendix

**Table 6:** Spread of COVID-19 and spatial wholesale spread (market *j* and *k*) (summary results)

	(1)	(2)	(3)	(4)	(5)
	Rice	Wheat	Gram	Tur	Urad
COVID-19^a^ (dummy)	0.056 (0.044)	0.048 (0.063)	0.242*** (0.059)	0.289*** (0.076)	− 0.069 (0.051)
Controls included	Yes	Yes	Yes	Yes	Yes
Monthly time dummies	Yes	Yes	Yes	Yes	Yes
Group-specific trend	Yes	Yes	No	No	Yes
Common time trend	No	No	Yes	Yes	No
Quadratic time trend	No	No	Yes	Yes	No
Observations	10,459	7437	10,114	9694	10,345
R-squared	0.345	0.297	0.039	0.011	0.318

	(6)	(7)	(8)	(9)	(10)
	Moong	Masoor	Milk	Onion	Potatoes
COVID-19^a^ (dummy)	0.221*** (0.059)	0.338*** (0.059)	0.018 (0.048)	− 0.161** (0.063)	0.096* (0.055)
Controls included	Yes	Yes	Yes	Yes	Yes
Monthly time dummies	Yes	Yes	Yes	Yes	Yes
Group-specific trend	Yes	Yes	No	No	Yes
Common time trend	No	No	Yes	Yes	No
Quadratic time trend	No	No	No	Yes	No
Observations	10,494	10,463	5760	10,529	10,540
R-squared	0.283	0.269	0.030	0.157	0.162

	(11)	(12)	(13)	(14)	(15)
	Tomatoes	Packaged oils	Tea	Salt	Sugar
COVID-19^a^ (dummy)	0.166*** (0.056)	− 0.051 (0.045)	0.103*** (0.027)	0.017 (0.038)	0.095** (0.047)
Controls included	Yes	Yes	Yes	Yes	Yes
Monthly time dummies	Yes	Yes	Yes	Yes	Yes
Group-specific trend	No	Yes	Yes	No	No
Common time trend	Yes	No	No	Yes	Yes
Quadratic time trend	Yes	No	No	Yes	Yes
Observations	10,483	10,529	7150	10,410	10,490
R-squared	0.045	0.285	0.358	0.029	0.016

Regressions are conducted for the period Jan 2019–June 2020. The dependent variable is the log-transformed absolute price difference in wholesale prices between market *j* and market *k*

*Significant at 10% level, **significant at 5% level, ***significant at 1% level

^§^Time-varying controls included: average rainfall in market *j* and market *k* and diesel prices in market *j* and market *k*

^a^COVID-19 takes a value of 1 if at least one market crosses the 100-caseload threshold. Nominal prices series have been deflated by the wholesale price index (2011–12 prices) and then all prices are log-transformed. Pulses: Gram (Chickpea), Tur (Pigeon pea), Urad (Black gram), Moong (Yellow lentils), Masoor (Red lentils). The full regression models are presented in Table A11 in the Online Appendix

**Table 7:** Spread of COVID-19 and market outcomes (continuous COVID-19 variable)

	(1)	(2)	(3)	(4)	(5)
	Retail prices	Wholesale prices	Vertical price dispersion	Spatial retail price dispersion	Spatial wholesale price dispersion
Storable					
Rice	0.010***	0.011***	− 0.004	0.008	0.010
Wheat	0.010***	0.012***	− 0.004	0.025**	0.000
Gram	0.018***	0.015***	0.041***	0.089***	0.094***
Tur	0.010***	0.007***	0.037***	0.107***	0.081**
Urad	0.017***	0.015***	0.026**	− 0.013	− 0.045
Moong	0.018***	0.017***	0.029***	0.094***	0.066***
Masoor	0.025***	0.024***	0.041***	0.084***	0.111***
Packaged oils	0.004**	0.004***	0.014	0.023**	− 0.011
Tea	0.008***	0.008***	0.018	0.015*	0.029***
Salt	0.011***	0.009**	0.012**	0.001	0.006
Sugar	0.012***	0.010***	0.018**	− 0.003	0.003
Perishable					
Milk	0.007**	0.010***	− 0.004	0.019**	0.013
Onion	− 0.112***	− 0.123***	− 0.079***	− 0.031**	− 0.059
Potatoes	0.025***	0.015***	0.037***	0.052***	0.038*
Tomatoes	− 0.051***	− 0.068***	− 0.007	0.071***	0.009

Regressions are conducted for the period Jan 2019–June 2020. Nominal prices series have been deflated by the wholesale price index (2011–12 prices) and then all prices are log-transformed. Vertical price dispersion is measured as a log of the absolute price difference between retail and wholesale prices for each market *i*. Spatial price dispersion is measured by the log-transformed absolute price difference between market *i* and market *j.* The main independent variable is a continuous variable that uses the inverse hyperbolic sine transformation of average daily COVID-19 caseloads. Standard errors are clustered by markets in parenthesis. Here, we present the coefficients of the main independent variable. Full regressions are presented in Tables A24–A28 in the Online Appendix. Pulses: Gram (Chickpea), Tur (Pigeon pea), Urad (Black gram), Moong (Yellow lentils), Masoor (Red lentils)

*Significant at 10% level, **significant at 5% level, ***significant at 1% level

### Results of robustness checks

*Excluding time-varying control variables correlated with the incidence of COVID-19 and dealing with time dependencies in the error term* In our main specification in Eqs. (1) and (2), we use a few continuous variables as controls. However, the nightlight index and diesel prices are correlated with the spread of COVID-19 (Table A12 in the Online Appendix). Therefore, we remove these two variables and re-estimate Eqs. (1) and (2) in Tables A13–A17 in the Online Appendix. We find that the interpretation of the coefficients for all our estimates remains stable. Further, in cross-sectional time-series data, there are often issues of autocorrelation. Therefore, like Aker (2010) we account for some time dependencies in unobserved stochastic error by re-estimating all equations by clustering the standard errors by quarters of the year. The results for our main outcome variables are presented in Tables A18–A22 in the Online Appendix. Both these robustness checks show that the results are very similar in terms of significance and signs of the coefficients to the estimates presented in Tables 2 and 3.

*Re-defining our treatment variable* One could question the definition of the treatment variable as we use a self-defined cut-off to capture markets where COVID-19 spread first, therefore we also re-define our treatment variable in three ways. First, we move the cut-off for our main estimates in Eq. (1) from 100 to 300 caseloads. For brevity, we present the summary results for cut-off at 300 caseloads in Table A23 in the Online Appendix. We find that the results are robust to changes in the threshold.

Second, we use a continuous variable, i.e., inverse hyperbolic sine transformation of average daily COVID-19 caseloads and re-estimate Eqs. (1, 2), and third, we use the timing of the national lockdown as a measure of supply shocks caused by the spread of COVID-19. The summary result with the continuous treatment variable is presented in Table 7. We do this exercise to validate the direction of association between market prices and the increase in COVID-19 caseloads. Like the previous findings, here too we find that as COVID-19 caseloads increased, market prices of commodities with longer shelf-life increased and prices of perishables declined in the period up to June 2020. However, the magnitude of the effect is smaller than the ones presented in Tables 2, 3, 4. Specifically, we find that on average a percentage increase in COVID-19 caseloads in each market, increased retail prices of cereals by 1%, pulses by 1–3%, potatoes by 3%, and processed items such as tea, salt, and sugar by about 1%. We find that retail prices of onions and tomatoes decreased by 11% and 5%, respectively. Further, we also see similar effects in the wholesale prices of commodities (Table 5). The highest vertical mark-ups were observed for pulses and potatoes. For every 1% increase in COVID-19 caseloads in each market, vertical price dispersion between retail and wholesale prices increased by 3–4% for pulses, and 4% for potatoes. Absolute price dispersion between retail and wholesale prices of onions fell by 8%. Similarly, we observe a statistically significant increase in spatial price dispersion in retail and wholesale markets for pulses, potatoes, and tomatoes.

Even using the timing of national lockdown as an additional robustness check shows very similar results to the ones presented in earlier sections, albeit the magnitude being larger for many commodities. We present the summary results of Eq. (3) in Table 8. Here we only present the coefficient of the interaction term ${\text{After}}_{t} \times {\text{COVID}}19_{ist}$ for brevity. In general, the results suggest that the average difference in prices after the lockdown was higher for most commodities in regions where COVID-19 spread. The exception is Onion and Tomatoes, where we see that the differential effect was lower.

**Table 8:** National lockdown and market prices (summary results)

	(1)	(2)
	Retail	Wholesale
Storable		
Rice	0.047**	0.076***
Wheat	0.000	0.000
Gram	0.038**	0.045*
Tur	0.000	0.000
Urad	0.065**	0.218***
Moong	0.053**	0.016
Masoor	0.087***	0.072***
Packaged oils	0.081***	0.070***
Tea	0.056***	− 0.002
Salt	0.156***	0.149***
Sugar	0.026**	− 0.059***
Perishable		
Milk	0.006	0.037*
Onion	− 0.265**	− 0.227*
Potatoes	0.233***	0.132**
Tomatoes	− 0.325***	− 0.369***

Table 8 presents the coefficient of the interaction term ${\text{After}}_{t} \times {\text{COVID}}19_{ist}$ for brevity. Full regression results are presented in Tables A29–A30 in the Online Appendix. Regressions are conducted for the period Jan 2019–June 2020. Nominal prices series have been deflated by the wholesale price index (2011–12 prices) and then all prices are log-transformed. Pulses: Gram (Chickpea), Tur (Pigeon pea), Urad (Black gram), Moong (Yellow lentils), Masoor (Red lentils)

*Significant at 10% level, **significant at 5% level, ***significant at 1% level

## Conclusion

The spread of COVID-19 in the first quarter of 2020 imposed several unprecedented national lockdowns that brought developed and developing economies to a standstill. Prohibition in the physical movement of humans and closure of public and private transportation, institutions, informal wet markets in developing countries, and shops, have had severe negative implications on economic activities, including the functioning of food supply chains. In this paper using data from urban markets in India and utilizing panel regression models with market fixed effects, we study how the spread of COVID-19 in the nascent phase of the pandemic affected market prices and how it distorted market outcomes by analyzing the price dispersions between markets in different regions and mark-ups between retail and wholesale markets.

Our results draw a more nuanced picture than earlier analysis focusing on short-term price effects only. We find that, as COVID-19 spread, prices in both retail and wholesale markets increased for commodities that have longer shelf-life such as cereals, pulses, and processed items (tea, packaged oils, sugar, and salt). However, prices of perishable items such as vegetables—specifically onions and tomatoes—declined substantially.

We think that these results are driven by both supply-side as well as demand-side factors. For non-perishable commodities that are storable for a longer period, prices could have increased due to increased consumer demand caused by panic buying, hoarding by traders, and due to supply-side bottlenecks in moving commodities from production hubs to the retail segment. On the other hand, perishables such as onions and tomatoes witnessed a decline in prices in India at the early onset of the pandemic. The decline in prices could be due to an oversupply of the produce because of the harvest of the ‘late Kharif’ and ‘Rabi’ season crop which occurred around the same time as when the COVID-19-related restrictions were imposed. It could also be due to a decline in institutional demand for these two items because of the closure of establishments such as hotels, restaurants, informal street-side eateries, etc., and due to the shift in consumer demand shifted from perishable items towards commodities with longer shelf-life.

Further, we find that market distortions increased significantly for most commodities. Pulses experienced large price distortions between markets as well as mark-ups between retail and wholesale prices. This could be related to increased transaction costs along the value chain. We, however, do not see any statistically significant price distortions in the market for rice and wheat, which are controlled by Government’s minimum support prices. Interestingly, we find that during the period of the study, the overall decline in onion prices in retail and wholesale markets also brought down existing price distortions in markets, unlike for tomatoes, wherein despite the fall in prices, market distortions increased. This result needs further attention and could be driven by a shift in the power relations between actors along the value chain.

Although we have not analyzed the welfare effects of the COVID-19 pandemic directly, these findings have strong implications for the welfare of consumers and farmers in India. From a policy point of view, the sharp drop in onion and tomato prices in the early stages of the pandemic, owed to unsold inventories and distressed sales caused by COVID-related increased transaction costs, highlights the urgent need for investment in cold storage, warehouses, and processing units in India to reduce loss of income by small farmers due to unexpected market and health shocks. Future research should focus on both short-term and long-term effects of COVID-19 on food prices disaggregated by the type of commodity. Further, while we find that prices of Onions and tomatoes fell at the onset of COVID-19, other studies should also analyze whether this pattern is also observable for other perishable commodities.

## Supplementary Information

Below is the link to the electronic supplementary material.

Supplementary file1 (DOCX 199 kb)

## Data Availability

Data are available on request.

## Footnotes

1 On 3rd February the Kerela Government declared COVID-19 a state calamity. Odisha and Delhi Government closed educational institutes, cinema halls, public swimming pools and gyms on March 13 and March 16, respectively.

2 Table A1 in the Appendix gives an overview of all the major COVID-19-related events reported by news sources between January–June 2020.

3 Curfew of the people.

4 Cereals (rice and wheat), pulses (gram, tur, urad,moong, lentil), persihable items (milk, potatoes, onion, tomatoes), processed items (packaged cooking oil, tea, salt and sugar).

5 https://consumeraffairs.nic.in/price-monitoring-cell/price-monitoring-cell.

6 https://eaindustry.nic.in/.

7 https://www.covid19india.org/.

8 https://chrsdata.eng.uci.edu/.

9 https://www.ppac.gov.in/content/5_1_ReportStudies.aspx.

10 As robustness check we also use move the cut-off from 100 to 300.

11 Measured in terms of absolute difference in retail and wholesale prices in market *i.* This is used as a measure of vertical integration between retail and wholesale prices of each commodity.

12 Event is described as daily COVID-19 caseloads crosses 100 for a specific state in which market i is located.

13 Media article.

14 https://www.business-standard.com/article/economy-policy/covid-19-impact-farmers-panic-as-onion-slips-to-season-s-lowest-of-rs-3-kg-120040601272_1.html.

15 As an additional robustness check, we estimate Eq. (1) using different specifications. First, we estimate a parismonius model with no controls then gradually we add adding the relevant covarites. These results are not shown for brevity. The results remain robust to these different specifications.
