# Supplementary material for: COVID-19 and distortions in urban food market in India
Source: Indian Econ Rev. 2022 May 30;57(1):133–64. doi: 10.1007/s41775-022-00130-3 (PMC9149335; doi:10.1007/s41775-022-00130-3)
Supplement: Supplementary file 1 — Supplementary file1 (DOCX 199 kb) [file 41775_2022_130_MOESM1_ESM.docx]

**Online Appendix**

**COVID-19 and distortions in urban food market in India**

**Table A1: Major events between January and June 2020**

| **Date** | **Major events (January-June 2020)** |
| --- | --- |
| 23-Jan-20 | China issues lockdown in several cities |
| 30-Jan-20 | WHO declared COVID-19 outbreak as a public health emergency |
| 30-Jan-20 | India reported the first COVID-19 case in Kerela |
| 31-Jan-20 | India airlifts citizens from China |
| 03-Feb-20 | Kerela Government declares coronavirus as a state calamity |
| 04-Feb-20 | India cancels existing visas for Chinese and foreigners who had visited China in the last two weeks |
| 08-Mar-20 | COVID-19 cases reported in 100 countries |
| 10-Mar-20 | 50 COVID 19 cases reported in India and the total cases doubled in just 4 days |
| 11-Mar-20 | WHO declared COVID-19 as a pandemic |
| 11-Mar-20 | India suspends all tourist visas |
| 12-Mar-20 | India records its first COVID-19 related death |
| 13-Mar-20 | Odisha Government closed educational institutes, cinema halls, public swimming pools and gyms |
| 14-Mar-20 | India crosses 100 COVID-19 confirmed cases |
| 16-Mar-20 | Delhi Government closes all gymnasiums, spas, night clubs and theatres until March 31. |
| 19-Mar-20 | India bans all incoming flights |
| 22-Mar-20 | India declares 14 hour voluntary lockdown called 'Janata curfew' |
| 22-Mar-20 | India suspends all international flights arriving and departing for at least one week |
| 25-Mar-20 | National lockdown imposed till April 14 |
| 26-Mar-20 | India witnesses huge exodus of migrant population from urban areas |
| 14-Apr-20 | National lockdown is extended for 21 days to May 3 |
| 28-Apr-20 | India records 1000 COVID-19 related deaths |
| 01-May-20 | National lockdown extended for two weeks starting May 4 |
| 17-May-20 | National lockdown extended till May 31 |
| 25-May-20 | Domestic flight services resume |
| 08-Jun-20 | India begins to unlock in a phased manner. Lockdown restrictions continued in containment zones. |

Source: Media reports^[[1]](#footnote-1)^

**Table A2: Region-wise timing of crossing 100 caseloads between March and June 2020**

| **State** | **Mar-20** | **Apr-20** | **May-20** | **Jun-20** |
| --- | --- | --- | --- | --- |
| Andaman and Nicobar | No | No | No | No |
| Andhra Pradesh | No | No | No | **Yes** |
| Arunachal Pradesh | No | No | No | No |
| Assam | No | No | No | **Yes** |
| Bihar | No | No | **Yes** | **Yes** |
| Chandigarh | No | No | No | No |
| Chattisgarh | No | No | No | No |
| Delhi | No | **Yes** | **Yes** | **Yes** |
| Goa | No | No | No | No |
| Gujarat | No | **Yes** | **Yes** | **Yes** |
| Haryana | No | No | No | **Yes** |
| Himachal Pradesh | No | No | No | No |
| Jammu and Kashmir | No | No | No | **Yes** |
| Jharkhand | No | No | No | No |
| Karnataka | No | No | No | **Yes** |
| Kerela | No | No | No | **Yes** |
| Madhya Pradesh | No | No | **Yes** | **Yes** |
| Maharashtra | No | **Yes** | **Yes** | **Yes** |
| Manipur | No | No | No | No |
| Meghalaya | No | No | No | No |
| Mizoram | No | No | No | No |
| Nagaland | No | No | No | No |
| Odisha | No | No | No | **Yes** |
| Puducherry | No | No | No | No |
| Punjab | No | No | No | **Yes** |
| Rajasthan | No | No | **Yes** | **Yes** |
| Sikkim | No | No | No | No |
| Tamil Nadu | No | No | **Yes** | **Yes** |
| Telangana | No | No | No | **Yes** |
| Tripura | No | No | No | No |
| Uttar Pradesh | No | No | **Yes** | **Yes** |
| Uttarakhand | No | No | No | No |
| West Bengal | No | No | **Yes** | **Yes** |

Source: COVID-19 India tracker. Data accessed 13.05.2021

**Table A3: Summary statistics**

|  | **(1)** | | | | **(2)** | | | | **(3)** | |
| --- | --- | --- | --- | --- | --- | --- | --- | --- | --- | --- |
|  | **Before event** | | | | **After event** | | | |  |  |
|  | **Mean** | **Standard Deviation** | **Min** | **Max** | **Mean** | **Standard Deviation** | **Min** | **Max** | **Mean difference** | **SE** |
| **1. Retail prices** | | | | | | | | | | |
| Rice (Rs/Kg) | 27.00 | 6.14 | 17.83 | 53.99 | 28.96 | 7.12 | 19.86 | 53.89 | 1.97*** | 0.53 |
| Wheat (Rs/Kg) | 22.89 | 5.73 | 13.17 | 51.66 | 23.11 | 5.63 | 14.25 | 36.68 | 0.22 | 0.5 |
| Gram (Rs/Kg) | 54.91 | 6.62 | 42.26 | 92.28 | 57.95 | 10.03 | 44.31 | 102.13 | 3.05*** | 0.59 |
| Tur (Rs/Kg) | 69.81 | 9.23 | 47.70 | 110.64 | 78.40 | 8.51 | 56.28 | 100.59 | 8.59*** | 0.79 |
| Urad (Rs/Kg) | 70.03 | 16.41 | 39.47 | 150.13 | 85.79 | 13.09 | 54.48 | 115.36 | 15.76*** | 1.37 |
| Moong (Rs/Kg) | 72.51 | 13.10 | 49.55 | 127.66 | 89.72 | 14.55 | 57.00 | 132.38 | 17.22*** | 1.12 |
| Masoor (Rs/Kg) | 54.17 | 8.33 | 37.01 | 98.93 | 64.02 | 11.24 | 45.96 | 109.06 | 9.85*** | 0.73 |
| Milk (Rs/Litre) | 36.93 | 6.29 | 19.13 | 83.89 | 38.83 | 6.32 | 18.72 | 61.28 | 1.90*** | 0.54 |
| Onion (Rs/Kg) | 28.54 | 18.92 | 5.02 | 123.80 | 18.86 | 10.44 | 8.38 | 113.45 | -9.68*** | 1.56 |
| Potatoes (Rs/Kg) | 17.24 | 6.06 | 5.89 | 41.52 | 22.43 | 5.30 | 13.41 | 34.31 | 5.20*** | 0.51 |
| Tomatoes (Rs/Kg) | 24.19 | 10.25 | 6.10 | 82.30 | 19.92 | 10.77 | 5.18 | 70.23 | -4.28*** | 0.87 |
| Packaged oil (Rs/Kg) | 101.31 | 11.37 | 76.67 | 139.59 | 107.99 | 10.74 | 85.08 | 139.50 | 6.68*** | 0.96 |
| Tea (Rs/Kg) | 178.74 | 39.52 | 75.50 | 385.58 | 185.42 | 37.35 | 100.59 | 297.87 | 6.67** | 3.36 |
| Salt (Rs/Kg) | 12.79 | 3.13 | 4.95 | 18.72 | 13.51 | 3.38 | 7.54 | 21.28 | 0.71*** | 0.27 |
| Sugar (Rs/Kg) | 32.37 | 2.76 | 27.55 | 47.54 | 33.25 | 2.33 | 28.85 | 42.55 | 0.89*** | 0.23 |
| **2. Wholesale prices** | | | | | | | | | | |
| Rice (Rs/Kg) | 24.25 | 5.70 | 16.26 | 52.33 | 26.05 | 6.88 | 18.16 | 51.01 | 1.81*** | 0.49 |
| Wheat (Rs/Kg) | 20.43 | 4.96 | 12.28 | 42.55 | 20.55 | 4.93 | 12.57 | 34.59 | 0.12 | 0.44 |
| Gram (Rs/Kg) | 49.63 | 6.09 | 36.18 | 93.68 | 52.10 | 10.07 | 38.24 | 102.13 | 2.47*** | 0.55 |
| Tur (Rs/Kg) | 64.34 | 8.44 | 42.43 | 100.19 | 71.39 | 7.72 | 50.58 | 100.59 | 7.06*** | 0.73 |
| Urad (Rs/Kg) | 64.05 | 15.10 | 36.18 | 138.73 | 78.41 | 12.38 | 51.00 | 103.94 | 14.36*** | 1.27 |
| Moong (Rs/Kg) | 66.99 | 12.14 | 36.18 | 117.91 | 82.75 | 13.53 | 51.13 | 117.35 | 15.76*** | 1.04 |
| Masoor (Rs/Kg) | 49.01 | 8.07 | 34.13 | 100.67 | 58.47 | 11.16 | 41.67 | 106.26 | 9.46*** | 0.72 |
| Milk (Rs/Litre) | 34.77 | 6.36 | 17.53 | 74.59 | 36.08 | 7.62 | 17.02 | 61.70 | 1.31* | 0.67 |
| Onion (Rs/Kg) | 23.42 | 16.67 | 2.02 | 103.34 | 13.72 | 11.10 | 3.47 | 113.45 | -9.70*** | 1.39 |
| Potatoes (Rs/Kg) | 13.36 | 5.58 | 2.50 | 35.51 | 17.31 | 5.30 | 6.47 | 34.99 | 3.95*** | 0.47 |
| Tomatoes (Rs/Kg) | 19.09 | 9.64 | 3.92 | 82.30 | 14.42 | 11.41 | 3.48 | 70.72 | -4.67*** | 0.83 |
| Packaged oil (Rs/Kg) | 94.06 | 10.16 | 67.33 | 138.68 | 100.84 | 9.26 | 79.59 | 131.86 | 6.78*** | 0.87 |
| Tea (Rs/Kg) | 159.67 | 40.24 | 83.68 | 384.95 | 167.02 | 42.88 | 92.38 | 297.87 | 7.35** | 3.73 |
| Salt (Rs/Kg) | 10.65 | 3.35 | 3.29 | 17.31 | 11.20 | 3.75 | 3.86 | 21.28 | 0.55* | 0.29 |
| Sugar (Rs/Kg) | 29.84 | 2.31 | 24.39 | 45.47 | 30.74 | 2.62 | 27.24 | 42.55 | 0.90*** | 0.2 |
| **3. Absolute price difference (retail and wholesale prices)** | | | | | | | | | | |
| Rice (Rs/Kg) | 2.77 | 1.54 | 0.00 | 11.12 | 2.93 | 1.70 | 0.00 | 8.94 | 0.15 | 0.13 |
| Wheat (Rs/Kg) | 2.45 | 1.44 | 0.00 | 12.93 | 2.55 | 1.57 | 0.00 | 9.94 | 0.1 | 0.13 |
| Gram (Rs/Kg) | 5.24 | 3.60 | 0.09 | 22.10 | 5.83 | 4.67 | 0.00 | 24.93 | 0.59* | 0.31 |
| Tur (Rs/Kg) | 5.40 | 3.63 | 0.00 | 28.21 | 6.97 | 4.74 | 0.00 | 22.98 | 1.57*** | 0.32 |
| Urad (Rs/Kg) | 5.90 | 4.49 | 0.00 | 35.87 | 7.39 | 5.78 | 0.00 | 28.32 | 1.49*** | 0.39 |
| Moong (Rs/Kg) | 5.44 | 3.91 | 0.00 | 32.34 | 7.04 | 5.44 | 0.00 | 25.80 | 1.60*** | 0.35 |
| Masoor (Rs/Kg) | 5.04 | 3.55 | 0.00 | 26.36 | 5.52 | 4.10 | 0.00 | 22.80 | 0.48 | 0.31 |
| Milk (Rs/Litre) | 2.38 | 1.90 | 0.00 | 18.14 | 2.54 | 1.94 | 0.00 | 8.51 | 0.16 | 0.2 |
| Onion (Rs/Kg) | 5.17 | 4.07 | 0.00 | 36.79 | 5.15 | 4.28 | 0.00 | 23.50 | -0.02 | 0.35 |
| Potatoes (Rs/Kg) | 3.83 | 2.22 | 0.00 | 15.87 | 5.09 | 3.51 | 0.00 | 16.73 | 1.26*** | 0.2 |
| Tomatoes (Rs/Kg) | 5.06 | 3.61 | 0.00 | 24.21 | 5.47 | 4.57 | 0.00 | 26.24 | 0.41 | 0.31 |
| Packaged oil (Rs/Kg) | 7.22 | 5.32 | 0.00 | 39.99 | 7.15 | 4.94 | 0.00 | 33.62 | -0.07 | 0.45 |
| Tea (Rs/Kg) | 19.00 | 15.22 | 0.00 | 218.12 | 19.12 | 15.93 | 0.00 | 81.34 | 0.12 | 1.41 |
| Salt (Rs/Kg) | 2.07 | 1.24 | 0.00 | 9.23 | 2.14 | 1.33 | 0.00 | 6.81 | 0.08 | 0.11 |
| Sugar (Rs/Kg) | 2.47 | 1.31 | 0.00 | 14.12 | 2.49 | 1.37 | 0.00 | 7.39 | 0.02 | 0.11 |
| **4. Other Control variables** | | | | | | | | | | |
| Night light (radiance/ pixel/ month) | 1.52 | 1.58 | 0.01 | 12.32 | 1.72 | 1.66 | 0.11 | 11.01 | 0.21** | 0.13 |
| Rainfall (mm) | 92.00 | 119.07 | 0.00 | 604.76 | 152.93 | 127.35 | 0.77 | 436.34 | 60.93*** | 10.03 |
| Diesel prices (Rs/litre) | 56.07 | 1.64 | 52.19 | 62.79 | 59.17 | 2.57 | 52.26 | 62.79 | 3.09*** | 0.15 |
| Observations |  |  |  |  |  |  |  |  | 2,053 |  |

Mean difference before and after the event. The event is described as daily COVID-19 caseloads cross 100 for a specific state in which market i is located. Pulses: Gram (Chickpea), Tur (Pigeon pea), Urad (Black gram), Moong (Yellow lentils), Masoor (Red lentils). * significant at 10% level, ** significant at 5% level, ***significant at 1% level. All price data are deflated by the wholesale price index at 2012 prices.

**Table A4:** **Spread of COVID-19 and retail prices (COVID-19 caseloads at state level)**

|  | **(1)** | **(2)** | **(3)** | **(4)** | **(5)** | **(6)** | **(7)** | **(8)** | **(9)** | **(10)** | **(11)** | **(12)** | **(13)** | **(14)** | **(15)** |  |
| --- | --- | --- | --- | --- | --- | --- | --- | --- | --- | --- | --- | --- | --- | --- | --- | --- |
|  | **Retail prices (log)** | | | | | | | | | | | | | | | |
|  | **Rice** | **Wheat** | **Gram** | **Tur** | **Urad** | **Moong** | **Masoor** | **Milk** | **Onion** | **Potatoes** | **Tomatoes** | **Packaged Oils** | **Tea** | **Salt** | **Sugar** |  |
| COVID 19^*^ (dummy) | 0.026** | 0.040*** | 0.043*** | 0.032** | 0.059** | 0.054*** | 0.083*** | 0.022*** | -0.416*** | 0.108*** | -0.172*** | 0.019** | 0.019 | 0.040*** | 0.030*** |  |
|  | (0.012) | (0.011) | (0.013) | (0.015) | (0.023) | (0.016) | (0.014) | (0.007) | (0.049) | (0.026) | (0.050) | (0.008) | (0.015) | (0.014) | (0.007) |  |
| Night light (log) | 0.038** | 0.030 | -0.011 | -0.006 | 0.021 | 0.012 | 0.006 | 0.000 | 0.014 | 0.044 | -0.032 | 0.020** | 0.004 | -0.009 | 0.010 |  |
|  | (0.016) | (0.019) | (0.011) | (0.014) | (0.022) | (0.014) | (0.016) | (0.009) | (0.032) | (0.029) | (0.066) | (0.009) | (0.014) | (0.020) | (0.007) |  |
| Rainfall (log) | 0.002 | 0.002 | 0.001 | 0.002 | 0.003 | 0.006*** | 0.005*** | 0.002** | -0.004 | -0.006* | -0.001 | 0.002* | 0.001 | 0.002 | 0.002* |  |
|  | (0.001) | (0.002) | (0.002) | (0.002) | (0.002) | (0.002) | (0.002) | (0.001) | (0.007) | (0.003) | (0.006) | (0.001) | (0.001) | (0.001) | (0.001) |  |
| Diesel price (log) | -0.113* | -0.170** | -0.152** | -0.387*** | -0.256** | -0.142 | 0.221*** | 0.080 | -1.114*** | -0.557*** | -1.575*** | -0.000 | 0.052 | 0.037 | -0.101** |  |
|  | (0.068) | (0.083) | (0.066) | (0.091) | (0.099) | (0.101) | (0.071) | (0.052) | (0.280) | (0.154) | (0.323) | (0.040) | (0.061) | (0.081) | (0.042) |  |
| Year fixed effects | Yes | Yes | Yes | Yes | Yes | Yes | Yes | Yes | Yes | Yes | Yes | Yes | Yes | Yes | Yes |  |
| Market fixed effects | Yes | Yes | Yes | Yes | Yes | Yes | Yes | Yes | Yes | Yes | Yes | Yes | Yes | Yes | Yes |  |
| Group-specific time trend | Yes | Yes | Yes | Yes | Yes | Yes | Yes | Yes | Yes | Yes | Yes | Yes | Yes | Yes | Yes |  |
| Monthly time dummies | Yes | Yes | Yes | Yes | Yes | Yes | Yes | Yes | Yes | Yes | Yes | Yes | Yes | Yes | Yes |  |
| State x monthly time dummies | Yes | Yes | Yes | Yes | Yes | Yes | Yes | Yes | Yes | Yes | Yes | Yes | Yes | Yes | Yes |  |
| Observations | 1,971 | 1,824 | 1,961 | 1,946 | 1,966 | 1,971 | 1,966 | 1,964 | 1,971 | 1,971 | 1,967 | 1,971 | 1,956 | 1,971 | 1,971 |  |
| R-squared | 0.694 | 0.596 | 0.451 | 0.753 | 0.842 | 0.841 | 0.701 | 0.755 | 0.899 | 0.793 | 0.658 | 0.772 | 0.504 | 0.552 | 0.469 |  |

Regressions are conducted for the period Jan 2019 to June 2020. ^*^ Daily COVID 19 caseloads cross 100 for a specific state in which market i is located. *Significant at 10% level, ** Significant at 5% level, ***Significant at 1% level. Nominal prices series have been deflated by the wholesale price index (2011-12 prices) and then all prices are log-transformed. Standard errors are clustered by markets in parenthesis. Pulses: Gram (Chickpea), Tur (Pigeon pea), Urad (Black gram), Moong (Yellow lentils), Masoor (Red lentils).

**Table A5: Spread of COVID-19 and wholesale prices (COVID-19 caseloads at state level)**

|  | **(1)** | **(2)** | **(3)** | **(4)** | **(5)** | **(6)** | **(7)** | **(8)** | **(9)** | **(10)** | **(11)** | **(12)** | **(13)** | **(14)** | **(15)** |
| --- | --- | --- | --- | --- | --- | --- | --- | --- | --- | --- | --- | --- | --- | --- | --- |
|  | **Wholesale prices (log)** | | | | | | | | | | | | | | |
|  | **Rice** | **Wheat** | **Gram** | **Tur** | **Urad** | **Moong** | **Masoor** | **Milk** | **Onion** | **Potatoes** | **Tomatoes** | **Packaged Oils** | **Tea** | **Salt** | **Sugar** |
| COVID 19^*^ (dummy) | 0.033*** | 0.049*** | 0.031*** | 0.020 | 0.052** | 0.047*** | 0.072*** | 0.031*** | -0.478*** | 0.077*** | -0.223*** | 0.017** | 0.021*** | 0.026* | 0.025*** |
|  | (0.010) | (0.010) | (0.011) | (0.014) | (0.020) | (0.014) | (0.011) | (0.009) | (0.055) | (0.028) | (0.054) | (0.008) | (0.008) | (0.014) | (0.006) |
| Night light (log) | 0.041** | 0.045** | -0.019 | -0.005 | 0.033 | 0.013 | 0.005 | -0.001 | 0.017 | 0.037 | -0.058 | 0.020* | -0.001 | -0.015 | 0.002 |
|  | (0.017) | (0.018) | (0.012) | (0.016) | (0.024) | (0.015) | (0.015) | (0.010) | (0.040) | (0.029) | (0.081) | (0.010) | (0.009) | (0.020) | (0.006) |
| Rainfall (log) | 0.003* | 0.002 | 0.002 | 0.003* | 0.004** | 0.008*** | 0.006*** | 0.002 | -0.002 | -0.005 | 0.008 | 0.003*** | 0.002 | 0.002 | 0.002** |
|  | (0.001) | (0.002) | (0.001) | (0.002) | (0.002) | (0.002) | (0.002) | (0.002) | (0.008) | (0.004) | (0.006) | (0.001) | (0.002) | (0.002) | (0.001) |
| Diesel price (log) | -0.111* | -0.172** | -0.112* | -0.335*** | -0.262*** | -0.160 | 0.238*** | 0.041 | -1.669*** | -0.641*** | -1.691*** | 0.016 | 0.082* | 0.062 | -0.112*** |
|  | (0.067) | (0.084) | (0.063) | (0.096) | (0.091) | (0.101) | (0.068) | (0.055) | (0.308) | (0.203) | (0.372) | (0.042) | (0.042) | (0.082) | (0.037) |
| Year fixed effects | Yes | Yes | Yes | Yes | Yes | Yes | Yes | Yes | Yes | Yes | Yes | Yes | Yes | Yes | Yes |
| Market fixed effects | Yes | Yes | Yes | Yes | Yes | Yes | Yes | Yes | Yes | Yes | Yes | Yes | Yes | Yes | Yes |
| Group-specific time trend | Yes | Yes | Yes | Yes | Yes | Yes | Yes | Yes | Yes | Yes | Yes | Yes | Yes | Yes | Yes |
| Monthly time dummies | Yes | Yes | Yes | Yes | Yes | Yes | Yes | Yes | Yes | Yes | Yes | Yes | Yes | Yes | Yes |
| State x monthly time dummies | Yes | Yes | Yes | Yes | Yes | Yes | Yes | Yes | Yes | Yes | Yes | Yes | Yes | Yes | Yes |
| Observations | 1,954 | 1,797 | 1,945 | 1,926 | 1,947 | 1,954 | 1,940 | 1,434 | 1,954 | 1,953 | 1,943 | 1,947 | 1,667 | 1,910 | 1,954 |
| R-squared | 0.706 | 0.595 | 0.460 | 0.737 | 0.849 | 0.842 | 0.744 | 0.755 | 0.897 | 0.822 | 0.670 | 0.774 | 0.606 | 0.615 | 0.506 |

Regressions are conducted for the period Jan 2019 to June 2020. ^*^ Daily COVID 19 caseloads cross 100 for a specific state in which market i is located. * Significant at 10% level, ** Significant at 5% level, ***Significant at 1% level. Nominal prices series have been deflated by the wholesale price index (2011-12 prices) and then all prices are log-transformed. Standard errors are clustered by markets in parenthesis. Pulses: Gram (Chickpea), Tur (Pigeon pea), Urad (Black gram), Moong (Yellow lentils), Masoor (Red lentils).

**Table A6: Spread of COVID-19 and retail prices (COVID-19 caseloads at district level)**

|  | **(1)** | **(2)** | **(3)** | **(4)** | **(5)** | **(6)** | **(7)** | **(8)** | **(9)** | **(10)** | **(11)** | **(12)** | **(13)** | **(14)** | **(15)** |
| --- | --- | --- | --- | --- | --- | --- | --- | --- | --- | --- | --- | --- | --- | --- | --- |
|  | **Retail prices (log)** | | | | | | | | | | | | | | |
|  | **Rice** | **Wheat** | **Gram** | **Tur** | **Urad** | **Moong** | **Masoor** | **Milk** | **Onion** | **Potatoes** | **Tomatoes** | **Packaged oil** | **Tea** | **Salt** | **Sugar** |
| COVID 19* (dummy) | 0.027* | 0.018 | 0.055*** | 0.007 | 0.047** | 0.050*** | 0.083*** | 0.024*** | -0.327*** | 0.109*** | -0.215*** | 0.019* | 0.024*** | 0.039*** | 0.037*** |
|  | (0.016) | (0.013) | (0.013) | (0.015) | (0.019) | (0.018) | (0.017) | (0.009) | (0.045) | (0.031) | (0.048) | (0.010) | (0.009) | (0.012) | (0.007) |
| Night light (log) | 0.009 | 0.007 | -0.017 | -0.027 | -0.007 | -0.035** | -0.024 | -0.007 | 0.047 | 0.018 |  | 0.033** | 0.016 | -0.018 | 0.013 |
|  | (0.014) | (0.024) | (0.013) | (0.021) | (0.031) | (0.015) | (0.021) | (0.012) | (0.044) | (0.040) |  | (0.013) | (0.017) | (0.026) | (0.010) |
| Rainfall (log) | 0.001 | 0.002 | 0.002 | 0.001 | -0.001 | 0.006*** | 0.007*** | 0.003* | -0.022*** | -0.004 | 0.000 | 0.002** | 0.003* | 0.003 | 0.002** |
|  | (0.002) | (0.002) | (0.001) | (0.002) | (0.003) | (0.002) | (0.002) | (0.001) | (0.008) | (0.004) | (0.008) | (0.001) | (0.002) | (0.002) | (0.001) |
| Diesel price (log) | -0.156** | -0.081 | -0.204*** | -0.245** | -0.216** | -0.149 | 0.221** | 0.050 | -1.669*** | -0.641*** |  | -0.013 | 0.034 | 0.067 | -0.135*** |
|  | (0.078) | (0.090) | (0.071) | (0.105) | (0.104) | (0.116) | (0.092) | (0.057) | (0.289) | (0.173) |  | (0.056) | (0.079) | (0.087) | (0.038) |
| Year fixed effects | Yes | Yes | Yes | Yes | Yes | Yes | Yes | Yes | Yes | Yes | Yes | Yes | Yes | Yes | Yes |
| Market fixed effects | Yes | Yes | Yes | Yes | Yes | Yes | Yes | Yes | Yes | Yes | Yes | Yes | Yes | Yes | Yes |
| Group-specific time trend | Yes | Yes | Yes | No | No | Yes | Yes | Yes | Yes | Yes | Yes | Yes | Yes | Yes | Yes |
| Common time trend | No | No | No | Yes | Yes | No | No | No | No | No | No | No | No | No | No |
| Monthly time dummies | Yes | Yes | Yes | Yes | Yes | Yes | Yes | Yes | Yes | Yes | Yes | Yes | Yes | Yes | Yes |
| State x monthly time dummies | Yes | Yes | Yes | Yes | Yes | Yes | Yes | Yes | Yes | Yes | Yes | Yes | Yes | Yes | Yes |
| Observations | 1,326 | 1,259 | 1,322 | 1,321 | 1,324 | 1,326 | 1,326 | 1,322 | 1,326 | 1,326 | 1,326 | 1,326 | 1,313 | 1,326 | 1,326 |
| R-squared | 0.708 | 0.712 | 0.526 | 0.645 | 0.757 | 0.848 | 0.716 | 0.693 | 0.900 | 0.785 | 0.622 | 0.770 | 0.533 | 0.628 | 0.512 |

Regressions are conducted for the period Jan 2019 to June 2020. ^*^ Daily COVID 19 caseloads cross 100 for a specific district in which market i is located. *Significant at 10% level, ** Significant at 5% level, ***Significant at 1% level. Nominal prices series have been deflated by the wholesale price index (2011-12 prices) and then all prices are log-transformed. Standard errors are clustered by markets in parenthesis. Pulses: Gram (Chickpea), Tur (Pigeon pea), Urad (Black gram), Moong (Yellow lentils), Masoor (Red lentils).

**Table A7: Spread of COVID-19 and wholesale prices (COVID-19 caseloads at district level)**

|  | **(1)** | **(2)** | **(3)** | **(4)** | **(5)** | **(6)** | **(7)** | **(8)** | **(9)** | **(10)** | **(11)** | **(12)** | **(13)** | **(14)** | **(15)** |
| --- | --- | --- | --- | --- | --- | --- | --- | --- | --- | --- | --- | --- | --- | --- | --- |
|  | **Wholesale prices (log)** | | | | | | | | | | | | | | |
|  | **Rice** | **Wheat** | **Gram** | **Tur** | **Urad** | **Moong** | **Masoor** | **Milk** | **Onion** | **Potatoes** | **Tomatoes** | **Packaged oil** | **Tea** | **Salt** | **Sugar** |
| COVID 19* (dummy) | 0.023 | 0.026** | 0.044*** | 0.002 | 0.037* | 0.032* | 0.063*** | 0.016 | -0.420*** | 0.059** | -0.127** | 0.014* | 0.025*** | 0.042*** | 0.030*** |
|  | (0.014) | (0.012) | (0.011) | (0.015) | (0.019) | (0.017) | (0.015) | (0.013) | (0.059) | (0.029) | (0.055) | (0.008) | (0.007) | (0.012) | (0.008) |
| Night light (log) | 0.012 | 0.042* | -0.032** | -0.028 | -0.011 | -0.025 | -0.028 | -0.014 | 0.037 | -0.003 | -0.106 | 0.024 | 0.002 | -0.045 | 0.004 |
|  | (0.016) | (0.023) | (0.016) | (0.022) | (0.031) | (0.016) | (0.020) | (0.010) | (0.060) | (0.046) | (0.137) | (0.015) | (0.018) | (0.031) | (0.009) |
| Rainfall (log) | 0.002 | 0.003 | 0.003 | 0.002 | 0.001 | 0.008*** | 0.009*** | 0.004 | -0.021** | -0.005 | 0.009 | 0.003*** | 0.003 | 0.003 | 0.002* |
|  | (0.002) | (0.002) | (0.002) | (0.002) | (0.003) | (0.002) | (0.002) | (0.003) | (0.009) | (0.005) | (0.009) | (0.001) | (0.002) | (0.002) | (0.001) |
| Diesel price (log) | -0.094 | -0.059 | -0.143** | -0.215* | -0.171 | -0.104 | 0.305*** | 0.123 | -2.258*** | -0.726*** | -2.197*** | 0.024 | 0.068 | 0.028 | -0.128*** |
|  | (0.085) | (0.088) | (0.062) | (0.110) | (0.115) | (0.119) | (0.084) | (0.084) | (0.344) | (0.198) | (0.378) | (0.050) | (0.066) | (0.088) | (0.043) |
| Year fixed effects | Yes | Yes | Yes | Yes | Yes | Yes | Yes | Yes | Yes | Yes | Yes | Yes | Yes | Yes | Yes |
| Market fixed effects | Yes | Yes | Yes | Yes | Yes | Yes | Yes | Yes | Yes | Yes | Yes | Yes | Yes | Yes | Yes |
| Group-specific time trend | Yes | Yes | Yes | Yes | Yes | Yes | Yes | Yes | Yes | Yes | Yes | Yes | Yes | Yes | Yes |
| Common time trend | No | No | No | No | Yes | No | No | No | No | No | No | No | No | No | No |
| Monthly time dummies | Yes | Yes | Yes | Yes | Yes | Yes | Yes | Yes | Yes | Yes | Yes | Yes | Yes | Yes | Yes |
| State x monthly time dummies | Yes | Yes | Yes | Yes | Yes | Yes | Yes | Yes | Yes | Yes | Yes | Yes | Yes | Yes | Yes |
| Observations | 1,310 | 1,245 | 1,306 | 1,304 | 1,306 | 1,310 | 1,298 | 937 | 1,310 | 1,309 | 1,310 | 1,305 | 1,092 | 1,267 | 1,310 |
| R-squared | 0.729 | 0.707 | 0.546 | 0.734 | 0.769 | 0.850 | 0.763 | 0.582 | 0.899 | 0.824 | 0.660 | 0.764 | 0.650 | 0.646 | 0.531 |

Regressions are conducted for the period Jan 2019 to June 2020. ^*^ Daily COVID 19 caseloads cross 100 for a specific district in which market i is located. *Significant at 10% level, ** Significant at 5% level, ***Significant at 1% level. Nominal prices series have been deflated by the wholesale price index (2011-12 prices) and then all prices are log-transformed. Standard errors are clustered by markets in parenthesis. Pulses: Gram (Chickpea), Tur (Pigeon pea), Urad (Black gram), Moong (Yellow lentils), Masoor (Red lentils).

**Table A8:** **Spread of COVID19 and vertical price dispersion between retail and wholesale prices (COVID-19 caseloads at state level)**

|  | **(1)** | **(2)** | **(3)** | **(4)** | **(5)** | **(6)** | **(7)** | **(8)** | **(9)** | **(10)** | **(11)** | **(12)** | **(13)** | **(14)** | **(15)** |  |
| --- | --- | --- | --- | --- | --- | --- | --- | --- | --- | --- | --- | --- | --- | --- | --- | --- |
|  | **Absolute price difference between retail and wholesale prices (log)** | | | | | | | | | | | | | | | |
|  | **Rice** | **Wheat** | **Gram** | **Tur** | **Urad** | **Moong** | **Masoor** | **Milk** | **Onion** | **Potatoes** | **Tomatoes** | **Packaged Oils** | **Tea** | **Salt** | **Sugar** |  |
| COVID-19* (dummy) | 0.002 | 0.009 | 0.127** | 0.186*** | 0.116 | 0.150** | 0.188*** | -0.047 | -0.310*** | 0.134** | -0.059 | 0.089** | 0.211*** | 0.064** | 0.058 |  |
|  | (0.045) | (0.043) | (0.050) | (0.063) | (0.080) | (0.068) | (0.066) | (0.056) | (0.063) | (0.058) | (0.067) | (0.045) | (0.067) | (0.030) | (0.046) |  |
| Night light (log) | 0.003 | -0.015 | 0.069 | -0.034 | -0.084 | 0.053 | 0.028 | 0.059 | -0.012 | 0.090 | 0.019 | -0.026 | -0.037 | 0.002 | 0.115** |  |
|  | (0.041) | (0.050) | (0.057) | (0.070) | (0.075) | (0.065) | (0.083) | (0.081) | (0.057) | (0.064) | (0.083) | (0.056) | (0.110) | (0.042) | (0.045) |  |
| Rain (log) | -0.004 | 0.001 | 0.004 | 0.001 | -0.011 | -0.011 | 0.002 | 0.008 | -0.004 | -0.007 | -0.024*** | -0.003 | 0.000 | 0.003 | 0.001 |  |
|  | (0.005) | (0.006) | (0.008) | (0.006) | (0.009) | (0.008) | (0.008) | (0.009) | (0.009) | (0.008) | (0.008) | (0.008) | (0.011) | (0.004) | (0.005) |  |
| Diesel price (log) | -0.192 | -0.348 | -0.305 | -0.851** | -0.328 | -0.021 | 0.082 | 0.010 | 0.736** | -0.297 | -0.545 | -0.385 | -1.264*** | 0.024 | -0.090 |  |
|  | (0.223) | (0.258) | (0.327) | (0.385) | (0.347) | (0.409) | (0.350) | (0.294) | (0.350) | (0.340) | (0.446) | (0.258) | (0.454) | (0.226) | (0.287) |  |
| Year fixed effects | Yes | Yes | Yes | Yes | Yes | Yes | Yes | Yes | Yes | Yes | Yes | Yes | Yes | Yes | Yes |  |
| Market fixed effects | Yes | Yes | Yes | Yes | Yes | Yes | Yes | Yes | Yes | Yes | Yes | Yes | Yes | Yes | Yes |  |
| Group-specific time trend | Yes | Yes | Yes | Yes | Yes | Yes | Yes | Yes | Yes | Yes | Yes | Yes | Yes | Yes | Yes |  |
| Monthly time dummies | Yes | Yes | Yes | Yes | Yes | Yes | Yes | Yes | Yes | Yes | Yes | Yes | Yes | Yes | Yes |  |
| State x monthly time dummies | Yes | Yes | Yes | Yes | Yes | Yes | Yes | Yes | Yes | Yes | Yes | Yes | Yes | Yes | Yes |  |
| Observations | 1,954 | 1,796 | 1,944 | 1,926 | 1,946 | 1,954 | 1,937 | 1,432 | 1,954 | 1,953 | 1,943 | 1,947 | 1,666 | 1,910 | 1,954 |  |
| R-squared | 0.401 | 0.475 | 0.530 | 0.545 | 0.499 | 0.525 | 0.421 | 0.461 | 0.659 | 0.461 | 0.447 | 0.425 | 0.553 | 0.533 | 0.431 |  |

Regressions are conducted for the period Jan 2019 to June 2020. ^*^ Daily COVID 19 caseloads cross 100 for a specific state in which market i is located. * Significant at 10% level, ** Significant at 5% level, ***Significant at 1% level. Nominal prices series have been deflated by the wholesale price index (2011-12 prices). The dependent variable is measured as a log of the absolute price difference between retail and wholesale prices. Standard errors are clustered by markets in parenthesis. Pulses: Gram (Chickpea), Tur (Pigeon pea), Urad (Black gram), Moong (Yellow lentils), Masoor (Red lentils).

**Table A9: Spread of COVID19 and vertical price dispersion between retail and wholesale prices (COVID-19 caseloads at district level)**

|  | **(1)** | **(2)** | **(3)** | **(4)** | **(5)** | **(6)** | **(7)** | **(8)** | **(9)** | **(10)** | **(11)** | **(12)** | **(13)** | **(14)** | **(15)** |
| --- | --- | --- | --- | --- | --- | --- | --- | --- | --- | --- | --- | --- | --- | --- | --- |
|  | **Absolute price difference between retail and wholesale prices (log)** | | | | | | | | | | | | | | |
|  | **Rice** | **Wheat** | **Gram** | **Tur** | **Urad** | **Moong** | **Masoor** | **Milk** | **Onion** | **Potatoes** | **Tomatoes** | **Packaged oil** | **Tea** | **Salt** | **Sugar** |
| COVID 19* (dummy) | 0.030 | -0.027 | 0.151** | 0.050 | 0.141** | 0.140* | 0.159*** | 0.121 | -0.213*** | 0.197*** | -0.028 | 0.029 | 0.040 | 0.052 | 0.092* |
|  | (0.049) | (0.039) | (0.069) | (0.066) | (0.070) | (0.082) | (0.058) | (0.093) | (0.055) | (0.070) | (0.057) | (0.048) | (0.044) | (0.033) | (0.053) |
| Night light (log) | -0.058 | -0.114 | 0.078 | 0.022 | -0.017 | -0.024 | 0.076 | -0.076 | 0.066 | 0.078 | 0.011 | 0.058 | 0.005 | 0.050 | 0.061 |
|  | (0.058) | (0.072) | (0.083) | (0.112) | (0.104) | (0.095) | (0.122) | (0.124) | (0.078) | (0.068) | (0.083) | (0.070) | (0.151) | (0.054) | (0.055) |
| Rainfall (log) | -0.005 | -0.006 | 0.017* | 0.010 | -0.010 | -0.005 | 0.005 | -0.015 | -0.013 | -0.001 | -0.024** | 0.013 | 0.015 | 0.005 | 0.011** |
|  | (0.006) | (0.006) | (0.009) | (0.009) | (0.012) | (0.008) | (0.011) | (0.010) | (0.013) | (0.009) | (0.010) | (0.009) | (0.016) | (0.005) | (0.005) |
| Diesel price (log) | -0.498** | -0.281 | -1.034** | -0.359 | -0.797* | -0.119 | -0.194 | -0.499 | 0.139 | -0.375 | -0.815** | -0.254 | -0.438 | 0.131 | -0.515* |
|  | (0.218) | (0.297) | (0.448) | (0.457) | (0.423) | (0.432) | (0.371) | (0.440) | (0.346) | (0.364) | (0.381) | (0.329) | (0.340) | (0.204) | (0.301) |
| Year fixed effects | Yes | Yes | Yes | Yes | Yes | Yes | Yes | Yes | Yes | Yes | Yes | Yes | Yes | Yes | Yes |
| Market fixed effects | Yes | Yes | Yes | Yes | Yes | Yes | Yes | Yes | Yes | Yes | Yes | Yes | Yes | Yes | Yes |
| Group-specific time trend | No | Yes | No | Yes | No | Yes | Yes | No | Yes | No | Yes | Yes | Yes | Yes |  |
| Common time trend | Yes | No | Yes | No | Yes | No | No | Yes | No | Yes | No | No | No | No | Yes |
| Square of common time trend | Yes | No | No | No | No | No | No | No | No | No | No | No | No | No | No |
| Monthly time dummies | Yes | Yes | Yes | Yes | Yes | Yes | Yes | Yes | Yes | Yes | Yes | Yes | Yes | Yes | Yes |
| State x monthly time dummies | Yes | Yes | Yes | Yes | Yes | Yes | Yes | Yes | Yes | Yes | Yes | Yes | Yes | Yes | Yes |
| Observations | 1,310 | 1,244 | 1,305 | 1,304 | 1,305 | 1,310 | 1,298 | 936 | 1,310 | 1,309 | 1,310 | 1,305 | 1,091 | 1,267 | 1,310 |
| R-squared | 0.146 | 0.519 | 0.169 | 0.591 | 0.245 | 0.596 | 0.488 | 0.162 | 0.685 | 0.217 | 0.242 | 0.482 | 0.686 | 0.639 | 0.105 |

Regressions are conducted for the period Jan 2019 to June 2020. ^*^ Daily COVID 19 caseloads cross 100 for a specific district in which market i is located. * Significant at 10% level, ** Significant at 5% level, ***Significant at 1% level. Nominal prices series have been deflated by the wholesale price index (2011-12 prices). The dependent variable is measured as a log of the absolute price difference between retail and wholesale prices. Standard errors are clustered by markets in parenthesis. Pulses: Gram (Chickpea), Tur (Pigeon pea), Urad (Black gram), Moong (Yellow lentils), Masoor (Red lentils).

**Table A10: Spread of COVID-19 and the spatial retail price difference (COVID-19 caseloads at state level)**

|  | **(1)** | **(2)** | **(3)** | **(4)** | **(5)** | **(6)** | **(7)** | **(8)** | **(9)** | **(10)** | **(11)** | **(12)** | **(13)** | **(14)** | **(15)** |
| --- | --- | --- | --- | --- | --- | --- | --- | --- | --- | --- | --- | --- | --- | --- | --- |
|  | **Absolute retail price difference between market j and k (log)** | | | | | | | | | | | | | | |
|  | **Rice** | **Wheat** | **Gram** | **Tur** | **Urad** | **Moong** | **Masoor** | **Milk** | **Onion** | **Potatoes** | **Tomatoes** | **Packaged Oils** | **Tea** | **Salt** | **Sugar** |
| COVID-19* (dummy) | 0.055 | 0.165*** | 0.562*** | 0.298*** | 0.046 | 0.343*** | 0.300*** | 0.016 | -0.151** | 0.146*** | 0.351*** | 0.097** | 0.027 | 0.027 | 0.027 |
|  | (0.044) | (0.053) | (0.052) | (0.068) | (0.047) | (0.060) | (0.055) | (0.032) | (0.065) | (0.053) | (0.055) | (0.047) | (0.030) | (0.035) | (0.045) |
| Rain (log) | 0.001 | -0.010 | -0.030*** | 0.010 | -0.016 | -0.015 | 0.034*** | -0.026*** | 0.042*** | -0.010 | 0.009 | 0.014* | 0.011** | 0.002 | -0.004 |
|  | (0.007) | (0.010) | (0.009) | (0.010) | (0.012) | (0.010) | (0.009) | (0.007) | (0.012) | (0.010) | (0.012) | (0.008) | (0.005) | (0.005) | (0.008) |
| Diesel (log) | -0.547* | -0.585* | -1.333*** | -2.732*** | -0.868** | -0.802* | 0.222 | 0.532** | 0.188 | 0.061 | -1.393*** | 0.084 | -0.048 | 0.069 | -0.114 |
|  | (0.307) | (0.313) | (0.371) | (0.448) | (0.388) | (0.464) | (0.383) | (0.208) | (0.454) | (0.391) | (0.463) | (0.355) | (0.179) | (0.236) | (0.357) |
| Monthly time dummies | Yes | Yes | Yes | Yes | Yes | Yes | Yes | Yes | Yes | Yes | Yes | Yes | Yes | Yes | Yes |
| Group specific trend | Yes | Yes | No | No | Yes | Yes | Yes | No | Yes | Yes | No | Yes | No | Yes | Yes |
| Common time trend | No | No | Yes | Yes | No | No | No | Yes | No | No | Yes | No | Yes | No | No |
| Quadratic time trend | | No | Yes | Yes | No | No | No | Yes | No | No | Yes | No | Yes | No | No |
| Year fixed effects | Yes | Yes | Yes | Yes | Yes | Yes | Yes | Yes | Yes | Yes | Yes | Yes | Yes | Yes | Yes |
| Market pair fixed effects | Yes | Yes | Yes | Yes | Yes | Yes | Yes | Yes | Yes | Yes | Yes | Yes | Yes | Yes | Yes |
| Observations | 10,868 | 7,842 | 10,547 | 10,200 | 10,867 | 11,046 | 11,001 | 10,655 | 11,059 | 11,083 | 11,031 | 11,157 | 10,503 | 10,211 | 10,827 |
| R-squared | 0.325 | 0.375 | 0.032 | 0.035 | 0.345 | 0.238 | 0.246 | 0.025 | 0.129 | 0.143 | 0.044 | 0.300 | 0.004 | 0.289 | 0.212 |

Regressions are conducted for the period Jan 20019 to June 2020. Dependent variable is the log transformed absolute price difference in retail prices between market j and market k*..* ^*^ COVID-19 takes a value of 1 if at least one market crosses the 100-caseload threshold. * Significant at 10% level, ** Significant at 5% level, ***Significant at 1% level. Nominal prices series have been deflated by the wholesale price index (2011-12 prices) and then all prices are log-transformed. Pulses: Gram (Chickpea), Tur (Pigeon pea), Urad (Black gram), Moong (Yellow lentils), Masoor (Red lentils).

**Table A11: Spread of COVID-19 and spatial wholesale price difference (COVID-19 caseloads at state level)**

|  | **(1)** | **(2)** | **(3)** | **(4)** | **(5)** | **(6)** | **(7)** | **(8)** | **(9)** | **(10)** | **(11)** | **(12)** | **(13)** | **(14)** | **(15)** |
| --- | --- | --- | --- | --- | --- | --- | --- | --- | --- | --- | --- | --- | --- | --- | --- |
|  | **Absolute wholesale price difference between market j and k (log)** | | | | | | | | | | | | | | |
|  | **Rice** | **Wheat** | **Gram** | **Tur** | **Urad** | **Moong** | **Masoor** | **Milk** | **Onion** | **Potatoes** | **Tomatoes** | **Packaged Oils** | **Tea** | **Salt** | **Sugar** |
| COVID-19* (dummy) | 0.056 | 0.048 | 0.242*** | 0.289*** | -0.069 | 0.221*** | 0.338*** | 0.018 | -0.161** | 0.096* | 0.166*** | -0.051 | 0.103*** | 0.017 | 0.095** |
|  | (0.044) | (0.063) | (0.059) | (0.076) | (0.051) | (0.059) | (0.059) | (0.048) | (0.063) | (0.055) | (0.056) | (0.045) | (0.027) | (0.038) | (0.047) |
| Rain (log) | -0.006 | -0.036*** | -0.034*** | -0.002 | 0.001 | -0.005 | 0.026** | -0.031*** | 0.036*** | 0.008 | 0.001 | 0.006 | -0.014*** | -0.007 | -0.001 |
|  | (0.009) | (0.012) | (0.010) | (0.012) | (0.012) | (0.010) | (0.010) | (0.010) | (0.012) | (0.011) | (0.012) | (0.011) | (0.005) | (0.005) | (0.008) |
| Diesel (log) | -0.566* | -0.420 | 0.881** | -2.173*** | -0.786* | -1.065** | 0.684* | 1.498*** | -0.889** | -0.493 | -0.866* | 0.250 | 0.127 | -0.697*** | -2.493*** |
|  | (0.306) | (0.437) | (0.405) | (0.518) | (0.408) | (0.476) | (0.401) | (0.398) | (0.446) | (0.437) | (0.486) | (0.384) | (0.201) | (0.250) | (0.354) |
| Monthly time dummies | Yes | Yes | Yes | Yes | Yes | Yes | Yes | Yes | Yes | Yes | Yes | Yes | Yes | Yes | Yes |
| Group specific trend | Yes | Yes | No | No | Yes | Yes | Yes | No | No | Yes | No | Yes | Yes | No | No |
| Common time trend | No | No | Yes | Yes | No | No | No | Yes | Yes | No | Yes | No | No | Yes | Yes |
| Quadratic time trend | No | No | Yes | Yes | No | No | No | No | Yes | No | Yes | No | No | Yes | Yes |
| Observations | 10,459 | 7,437 | 10,114 | 9,694 | 10,345 | 10,494 | 10,463 | 5,760 | 10,529 | 10,540 | 10,483 | 10,529 | 7,150 | 10,410 | 10,490 |
| R-squared | 0.345 | 0.297 | 0.039 | 0.011 | 0.318 | 0.283 | 0.269 | 0.030 | 0.157 | 0.162 | 0.045 | 0.285 | 0.358 | 0.029 | 0.016 |

Regressions are conducted for the period Jan 20019 to June 2020. Dependent variable is the log transformed absolute price difference in wholesale prices between market j and market k*.* ^§^ Time-varying controls included: average rainfall in market j and market k and diesel prices in market j and market k *.* ^*^ COVID-19 takes a value of 1 if at least one market crosses the 100-caseload threshold. * Significant at 10% level, ** Significant at 5% level, ***Significant at 1% level. Nominal prices series have been deflated by the wholesale price index (2011-12 prices) and then all prices are log-transformed. Pulses: Gram (Chickpea), Tur (Pigeon pea), Urad (Black gram), Moong (Yellow lentils), Masoor (Red lentils).

**Table A12: Correlation matrix**

|  | COVID-19 caseload^a^ (dummy) | COVID-19 caseloads^b^ (continuous) | Night light (log) | Diesel (log) |
| --- | --- | --- | --- | --- |
| COVID-19 caseload^a^ (dummy) | 1 |  |  |  |
| COVID-19 caseloads^b^ (continuous) | 0.775*** | 1 |  |  |
| Night light (log) | 0.055*** | 0.112*** | 1 |  |
| Diesel (log) | 0.419*** | 0.318*** | -0.151*** | 1 |

^a^ Daily COVID 19 caseloads cross 100 for a specific state in which market i is located. ^b^ Inverse hyperbolic sine transformation of the average daily COVID-19 caseloads. * Significant at 10% level, ** Significant at 5% level, ***Significant at 1% level.

**Table A13: Estimates on retail prices (robustness check: excluding night life and diesel prices)**

|  | **(1)** | **(2)** | **(3)** | **(4)** | **(5)** | **(6)** | **(7)** | **(8)** | **(9)** | **(10)** | **(11)** | **(12)** | **(13)** | **(14)** | **(15)** |
| --- | --- | --- | --- | --- | --- | --- | --- | --- | --- | --- | --- | --- | --- | --- | --- |
|  | **Rice** | **Wheat** | **Gram** | **Tur** | **Urad** | **Moong** | **Masoor** | **Milk** | **Onion** | **Potatoes** | **Tomatoes** | **Packaged Oils** | **Tea** | **Salt** | **Sugar** |
| COVID 19 ^a^ (dummy) | 0.017* | 0.026*** | 0.032*** | 0.002 | 0.039** | 0.043*** | 0.100*** | 0.028*** | -0.502*** | 0.065*** | -0.294*** | 0.019*** | 0.023 | 0.043*** | 0.022*** |
|  | (0.010) | (0.009) | (0.010) | (0.013) | (0.020) | (0.013) | (0.012) | (0.006) | (0.038) | (0.024) | (0.043) | (0.007) | (0.015) | (0.012) | (0.006) |
| Rainfall (log) | 0.001 | 0.001 | 0.001 | 0.002 | 0.002 | 0.006*** | 0.005*** | 0.002** | -0.006 | -0.008** | -0.002 | 0.001 | 0.001 | 0.002* | 0.001 |
|  | (0.002) | (0.002) | (0.002) | (0.002) | (0.002) | (0.002) | (0.002) | (0.001) | (0.007) | (0.003) | (0.006) | (0.001) | (0.001) | (0.001) | (0.001) |
| Year fixed effects | Yes | Yes | Yes | Yes | Yes | Yes | Yes | Yes | Yes | Yes | Yes | Yes | Yes | Yes | Yes |
| Market fixed effects | Yes | Yes | Yes | Yes | Yes | Yes | Yes | Yes | Yes | Yes | Yes | Yes | Yes | Yes | Yes |
| Group-specific time trend | Yes | Yes | Yes | Yes | Yes | Yes | Yes | Yes | Yes | Yes | Yes | Yes | Yes | Yes | Yes |
| Monthly time dummies | Yes | Yes | Yes | Yes | Yes | Yes | Yes | Yes | Yes | Yes | Yes | Yes | Yes | Yes | Yes |
| State x monthly time dummies | Yes | Yes | Yes | Yes | Yes | Yes | Yes | Yes | Yes | Yes | Yes | Yes | Yes | Yes | Yes |
| Observations | 1,971 | 1,824 | 1,961 | 1,946 | 1,966 | 1,971 | 1,966 | 1,964 | 1,971 | 1,971 | 1,967 | 1,971 | 1,956 | 1,971 | 1,971 |
| R-squared | 0.690 | 0.593 | 0.448 | 0.747 | 0.840 | 0.841 | 0.699 | 0.755 | 0.897 | 0.790 | 0.649 | 0.770 | 0.504 | 0.552 | 0.464 |

Regressions are conducted for the period Jan 2019 to June 2020. ^a^ Daily COVID 19 caseloads cross 100 for a specific state in which market i is located. * Significant at 10% level, ** Significant at 5% level, ***Significant at 1% level. Nominal prices series have been deflated by the wholesale price index (2011-12 prices) and then all prices are log-transformed. Standard errors are clustered by markets in parenthesis. Pulses: Gram (Chickpea), Tur (Pigeon pea), Urad (Black gram), Moong (Yellow lentils), Masoor (Red lentils).

**Table A14: Estimates on wholesale prices (robustness check: excluding night life and diesel prices)**

|  | **(1)** | **(2)** | **(3)** | **(4)** | **(5)** | **(6)** | **(7)** | **(8)** | **(9)** | **(10)** | **(11)** | **(12)** | **(13)** | **(14)** | **(15)** |
| --- | --- | --- | --- | --- | --- | --- | --- | --- | --- | --- | --- | --- | --- | --- | --- |
|  | **Rice** | **Wheat** | **Gram** | **Tur** | **Urad** | **Moong** | **Masoor** | **Milk** | **Onion** | **Potatoes** | **Tomatoes** | **Packaged Oils** | **Tea** | **Salt** | **Sugar** |
| COVID 19 ^a^ (dummy) | 0.025*** | 0.034*** | 0.023*** | -0.006 | 0.032* | 0.034*** | 0.090*** | 0.034*** | -0.606*** | 0.028 | -0.354*** | 0.018*** | 0.028*** | 0.030** | 0.017*** |
|  | (0.009) | (0.008) | (0.009) | (0.012) | (0.017) | (0.012) | (0.010) | (0.008) | (0.045) | (0.022) | (0.046) | (0.006) | (0.008) | (0.013) | (0.005) |
| Rainfall (log) | 0.002 | 0.001 | 0.002 | 0.002 | 0.003** | 0.008*** | 0.006*** | 0.002 | -0.004 | -0.006 | 0.007 | 0.002*** | 0.002 | 0.003* | 0.002** |
|  | (0.002) | (0.002) | (0.001) | (0.002) | (0.002) | (0.002) | (0.002) | (0.002) | (0.008) | (0.005) | (0.007) | (0.001) | (0.002) | (0.002) | (0.001) |
| Year fixed effects | Yes | Yes | Yes | Yes | Yes | Yes | Yes | Yes | Yes | Yes | Yes | Yes | Yes | Yes | Yes |
| Market fixed effects | Yes | Yes | Yes | Yes | Yes | Yes | Yes | Yes | Yes | Yes | Yes | Yes | Yes | Yes | Yes |
| Group-specific time trend | Yes | Yes | Yes | Yes | Yes | Yes | Yes | Yes | Yes | Yes | Yes | Yes | Yes | Yes | Yes |
| Monthly time dummies | Yes | Yes | Yes | Yes | Yes | Yes | Yes | Yes | Yes | Yes | Yes | Yes | Yes | Yes | Yes |
| State x monthly time dummies | Yes | Yes | Yes | Yes | Yes | Yes | Yes | Yes | Yes | Yes | Yes | Yes | Yes | Yes | Yes |
| Observations | 1,954 | 1,797 | 1,945 | 1,926 | 1,947 | 1,954 | 1,940 | 1,434 | 1,954 | 1,953 | 1,943 | 1,947 | 1,667 | 1,910 | 1,954 |
| R-squared | 0.703 | 0.589 | 0.458 | 0.733 | 0.847 | 0.841 | 0.741 | 0.755 | 0.894 | 0.819 | 0.662 | 0.773 | 0.605 | 0.615 | 0.503 |

Regressions are conducted for the period Jan 2019 to June 2020. ^a^ Daily COVID 19 caseloads cross 100 for a specific state in which market i is located. * Significant at 10% level, ** Significant at 5% level, ***Significant at 1% level. Nominal prices series have been deflated by the wholesale price index (2011-12 prices) and then all prices are log-transformed. Standard errors are clustered by markets in parenthesis. Pulses: Gram (Chickpea), Tur (Pigeon pea), Urad (Black gram), Moong (Yellow lentils), Masoor (Red lentils).

**Table A15: Absolute price difference between retail and wholesale prices (robustness check: excluding night light and diesel prices)**

|  | **(1)** | **(2)** | **(3)** | **(4)** | **(5)** | **(6)** | **(7)** | **(8)** | **(9)** | **(10)** | **(11)** | **(12)** | **(13)** | **(14)** | **(15)** |
| --- | --- | --- | --- | --- | --- | --- | --- | --- | --- | --- | --- | --- | --- | --- | --- |
|  | **Absolute price difference between retail and wholesale prices (log)** | | | | | | | | | | | | | | |
|  | **Rice** | **Wheat** | **Gram** | **Tur** | **Urad** | **Moong** | **Masoor** | **Milk** | **Onion** | **Potatoes** | **Tomatoes** | **Packaged Oils** | **Tea** | **Salt** | **Sugar** |
| COVID-19 ^a^ (dummy) | -0.012 | -0.019 | 0.103** | 0.119** | 0.091 | 0.148** | 0.194*** | -0.047 | -0.253*** | 0.111** | -0.101* | 0.059 | 0.115** | 0.066** | 0.050 |
|  | (0.039) | (0.036) | (0.047) | (0.053) | (0.069) | (0.059) | (0.058) | (0.045) | (0.047) | (0.048) | (0.053) | (0.041) | (0.052) | (0.027) | (0.039) |
| Rain (log) | -0.004 | 0.001 | 0.002 | 0.000 | -0.010 | -0.012 | 0.002 | 0.006 | -0.002 | -0.009 | -0.025*** | -0.003 | -0.001 | 0.003 | -0.002 |
|  | (0.005) | (0.006) | (0.007) | (0.007) | (0.009) | (0.007) | (0.008) | (0.009) | (0.009) | (0.008) | (0.008) | (0.008) | (0.010) | (0.004) | (0.005) |
| Year fixed effects | Yes | Yes | Yes | Yes | Yes | Yes | Yes | Yes | Yes | Yes | Yes | Yes | Yes | Yes | Yes |
| Market fixed effects | Yes | Yes | Yes | Yes | Yes | Yes | Yes | Yes | Yes | Yes | Yes | Yes | Yes | Yes | Yes |
| Group-specific time trend | Yes | Yes | Yes | Yes | Yes | Yes | Yes | Yes | Yes | Yes | Yes | Yes | Yes | Yes | Yes |
| Monthly time dummies | Yes | Yes | Yes | Yes | Yes | Yes | Yes | Yes | Yes | Yes | Yes | Yes | Yes | Yes | Yes |
| State x monthly time dummies | Yes | Yes | Yes | Yes | Yes | Yes | Yes | Yes | Yes | Yes | Yes | Yes | Yes | Yes | Yes |
| Observations | 1,954 | 1,796 | 1,944 | 1,926 | 1,946 | 1,954 | 1,937 | 1,432 | 1,954 | 1,953 | 1,943 | 1,947 | 1,666 | 1,910 | 1,954 |
| R-squared | 0.401 | 0.474 | 0.529 | 0.542 | 0.498 | 0.524 | 0.421 | 0.461 | 0.657 | 0.459 | 0.446 | 0.424 | 0.549 | 0.533 | 0.428 |

Regressions are conducted for the period Jan 2019 to June 2020. ^a^ Daily COVID 19 caseloads cross 100 for a specific state in which market i is located.* Significant at 10% level, ** Significant at 5% level, ***Significant at 1% level. Nominal prices series have been deflated by the wholesale price index (2011-12 prices). Dependent variable is measured as absolute price difference between retail and wholesale prices (Rs per unit). Standard errors are clustered by markets in parenthesis. Pulses: Gram (Chickpea), Tur (Pigeon pea), Urad (Black gram), Moong (Yellow lentils), Masoor (Red lentils).

**Table A16: Spread of COVID-19 and spatial retail price difference between markets (robustness check: excluding diesel prices)**

|  | **(1)** | **(2)** | **(3)** | **(4)** | **(5)** | **(6)** | **(7)** | **(8)** | **(9)** | **(10)** | **(11)** | **(12)** | **(13)** | **(14)** | **(15)** |
| --- | --- | --- | --- | --- | --- | --- | --- | --- | --- | --- | --- | --- | --- | --- | --- |
|  | **Absolute retail price difference between market j and k (log)** | | | | | | | | | | | | | | |
|  | **Rice** | **Wheat** | **Gram** | **Tur** | **Urad** | **Moong** | **Masoor** | **Milk** | **Onion** | **Potatoes** | **Tomatoes** | **Packaged Oils** | **Tea** | **Salt** | **Sugar** |
| COVID-19 ^a^ (dummy) | 0.023 | 0.125*** | 0.517*** | 0.179*** | -0.006 | 0.296*** | 0.313*** | 0.032 | -0.141*** | 0.149*** | 0.306*** | 0.102*** | 0.026 | 0.031 | 0.020 |
|  | (0.040) | (0.046) | (0.050) | (0.064) | (0.042) | (0.055) | (0.051) | (0.031) | (0.056) | (0.046) | (0.054) | (0.039) | (0.029) | (0.031) | (0.041) |
| Rain (log) | 0.000 | -0.010 | -0.031*** | 0.010 | -0.017 | -0.016 | 0.035*** | -0.026*** | 0.042*** | -0.010 | 0.008 | 0.014* | 0.011** | 0.002 | -0.005 |
|  | (0.007) | (0.010) | (0.009) | (0.010) | (0.012) | (0.010) | (0.009) | (0.007) | (0.012) | (0.010) | (0.011) | (0.008) | (0.005) | (0.005) | (0.008) |
| Monthly time dummies | Yes | Yes | Yes | Yes | Yes | Yes | Yes | Yes | Yes | Yes | Yes | Yes | Yes | Yes | Yes |
| Group specific trend | Yes | Yes | No | No | Yes | Yes | Yes | No | Yes | Yes | No | Yes | No | Yes | Yes |
| Common time trend | No | No | Yes | Yes | No | No | No | Yes | No | No | Yes | No | Yes | No | No |
| Quadratic time trend | No | No | Yes | Yes | No | No | No | Yes | No | No | Yes | No | Yes | No | No |
| Year fixed effects | Yes | Yes | Yes | Yes | Yes | Yes | Yes | Yes | Yes | Yes | Yes | Yes | Yes | Yes | Yes |
| Market pair fixed effects | Yes | Yes | Yes | Yes | Yes | Yes | Yes | Yes | Yes | Yes | Yes | Yes | Yes | Yes | Yes |
| Observations | 10,868 | 7,842 | 10,547 | 10,200 | 10,867 | 11,046 | 11,001 | 10655 | 11,059 | 11,083 | 11,031 | 11,157 | 10,503 | 10,211 | 10,827 |
| R-squared | 0.324 | 0.374 | 0.031 | 0.031 | 0.344 | 0.237 | 0.246 | 0.025 | 0.236 | 0.143 | 0.043 | 0.300 | 0.004 | 0.288 | 0.212 |

Regressions are conducted for the period Jan 20019 to June 2020. Dependent variable is the log transformed absolute price difference between market *i* and market *j.* ^a^ COVID-19 takes a value of 1 if at least one market crosses the 100-caseload threshold. * Significant at 10% level, ** Significant at 5% level, ***Significant at 1% level. Nominal prices series have been deflated by the wholesale price index (2011-12 prices) and then all prices are log-transformed. Pulses: Gram (Chickpea), Tur (Pigeon pea), Urad (Black gram), Moong (Yellow lentils), Masoor (Red lentils).

**Table A17: Spread of COVID-19 and wholesale spatial price difference between markets (robustness check: excluding diesel prices)**

|  | **(1)** | **(2)** | **(3)** | **(4)** | **(5)** | **(6)** | **(7)** | **(8)** | **(9)** | **(10)** | **(11)** | **(12)** | **(13)** | **(14)** | **(15)** |
| --- | --- | --- | --- | --- | --- | --- | --- | --- | --- | --- | --- | --- | --- | --- | --- |
|  | **Absolute wholesale price difference between market j and k (log)** | | | | | | | | | | | | | | |
|  | **Rice** | **Wheat** | **Gram** | **Tur** | **Urad** | **Moong** | **Masoor** | **Milk** | **Onion** | **Potatoes** | **Tomatoes** | **Packaged Oils** | **Tea** | **Salt** | **Sugar** |
| COVID-19^a^ (dummy) | 0.024 | 0.021 | 0.269*** | 0.201*** | -0.113** | 0.161*** | 0.377*** | 0.036 | -0.211*** | 0.068 | 0.140** | -0.036 | 0.110*** | 0.051* | 0.026 |
|  | (0.039) | (0.056) | (0.057) | (0.071) | (0.044) | (0.055) | (0.053) | (0.048) | (0.058) | (0.049) | (0.055) | (0.041) | (0.024) | (0.028) | (0.046) |
| Rain (log) | -0.007 | -0.037*** | -0.033*** | -0.003 | 0.000 | -0.006 | 0.027*** | -0.031*** | 0.035*** | 0.007 | 0.000 | 0.006 | -0.014*** | -0.005 | -0.002 |
|  | (0.009) | (0.012) | (0.010) | (0.012) | (0.012) | (0.010) | (0.010) | (0.010) | (0.011) | (0.011) | (0.012) | (0.011) | (0.005) | (0.005) | (0.008) |
| Monthly time dummies | Yes | Yes | Yes | Yes | Yes | Yes | Yes | Yes | Yes | Yes | Yes | Yes | Yes | Yes | Yes |
| Group specific trend | Yes | Yes | No | No | Yes | Yes | Yes | No | Yes | Yes | No | Yes | Yes | No | No |
| Common time trend | No | No | Yes | Yes | No | No | No | Yes | No | No | Yes | No | No | Yes | Yes |
| Quadratic time trend | No | No | Yes | Yes | No | No | No | Yes | No | No | Yes | No | No | No | Yes |
| Year fixed effects | Yes | Yes | Yes | Yes | Yes | Yes | Yes | Yes | Yes | Yes | Yes | Yes | Yes | Yes | Yes |
| Market pair fixed effects | Yes | Yes | Yes | Yes | Yes | Yes | Yes | Yes | Yes | Yes | Yes | Yes | Yes | Yes | Yes |
| Observations | 10,459 | 7,437 | 10,114 | 9,694 | 10,345 | 10,494 | 10,463 | 5,760 | 10,529 | 10,540 | 10,483 | 10,529 | 7,150 | 10,410 | 10,490 |
| R-squared | 0.345 | 0.296 | 0.038 | 0.008 | 0.318 | 0.282 | 0.268 | 0.028 | 0.157 | 0.162 | 0.045 | 0.285 | 0.358 | 0.028 | 0.011 |

Regressions are conducted for the period Jan 20019 to June 2020. Dependent variable is the log transformed absolute price difference between market *j* and market k*.* ^a^ COVID-19 takes a value of 1 if at least one market crosses the 100-caseload threshold.* Significant at 10% level, ** Significant at 5% level, ***Significant at 1% level. Nominal prices series have been deflated by the wholesale price index (2011-12 prices) and then all prices are log-transformed. Pulses: Gram (Chickpea), Tur (Pigeon pea), Urad (Black gram), Moong (Yellow lentils), Masoor (Red lentils).

**Table A18: Estimates on retail prices (robustness check: standard errors clustered by quarters)**

|  | **(1)** | **(2)** | **(3)** | **(4)** | **(5)** | **(6)** | **(7)** | **(8)** | **(9)** | **(10)** | **(11)** | **(12)** | **(13)** | **(14)** | **(15)** |  |
| --- | --- | --- | --- | --- | --- | --- | --- | --- | --- | --- | --- | --- | --- | --- | --- | --- |
|  | **Retail prices (log)** | | | | | | | | | | | | | | | |
|  | **Rice** | **Wheat** | **Gram** | **Tur** | **Urad** | **Moong** | **Masoor** | **Milk** | **Onion** | **Potatoes** | **Tomatoes** | **Packaged Oils** | **Tea** | **Salt** | **Sugar** |  |
| COVID 19 ^a^ (dummy) | 0.017 | 0.026** | 0.032 | 0.002 | 0.039 | 0.043 | 0.100** | 0.028** | -0.502** | 0.065* | -0.294** | 0.019** | 0.023** | 0.043** | 0.022 |  |
|  | (0.014) | (0.009) | (0.023) | (0.024) | (0.028) | (0.033) | (0.028) | (0.010) | (0.128) | (0.030) | (0.078) | (0.007) | (0.008) | (0.013) | (0.015) |  |
| Rainfall (log) | 0.001 | 0.001 | 0.001 | 0.002 | 0.002* | 0.006*** | 0.005*** | 0.002 | -0.006 | -0.008 | -0.002 | 0.001 | 0.001 | 0.002 | 0.001** |  |
|  | (0.001) | (0.002) | (0.001) | (0.001) | (0.001) | (0.001) | (0.001) | (0.002) | (0.009) | (0.005) | (0.005) | (0.001) | (0.001) | (0.001) | (0.000) |  |
| Year fixed effects | Yes | Yes | Yes | Yes | Yes | Yes | Yes | Yes | Yes | Yes | Yes | Yes | Yes | Yes | Yes |  |
| Market fixed effects | Yes | Yes | Yes | Yes | Yes | Yes | Yes | Yes | Yes | Yes | Yes | Yes | Yes | Yes | Yes |  |
| Group-specific time trend | Yes | Yes | Yes | Yes | Yes | Yes | Yes | Yes | Yes | Yes | Yes | Yes | Yes | Yes | Yes |  |
| Monthly time dummies | Yes | Yes | Yes | Yes | Yes | Yes | Yes | Yes | Yes | Yes | Yes | Yes | Yes | Yes | Yes |  |
| State x monthly time dummies | Yes | Yes | Yes | Yes | Yes | Yes | Yes | Yes | Yes | Yes | Yes | Yes | Yes | Yes | Yes |  |
| Observations | 1,971 | 1,824 | 1,961 | 1,946 | 1,966 | 1,971 | 1,966 | 1,964 | 1,971 | 1,971 | 1,967 | 1,971 | 1,956 | 1,971 | 1,971 |  |
| R-squared | 0.952 | 0.952 | 0.866 | 0.858 | 0.911 | 0.905 | 0.882 | 0.956 | 0.908 | 0.907 | 0.783 | 0.931 | 0.936 | 0.958 | 0.884 |  |

Regressions are conducted for the period Jan 2019 to June 2020. ^a^ Daily COVID 19 caseloads cross 100 for a specific state in which market i is located.* Significant at 10% level, ** Significant at 5% level, ***Significant at 1% level. Nominal prices series have been deflated by the wholesale price index (2011-12 prices) and then all prices are log-transformed. Standard errors are clustered by quarters of the year in parenthesis. Pulses: Gram (Chickpea), Tur (Pigeon pea), Urad (Black gram), Moong (Yellow lentils), Masoor (Red lentils).

**Table A19: Estimates on wholesale prices (robustness check: standard errors clustered by quarters)**

|  | **(1)** | **(2)** | **(3)** | **(4)** | **(5)** | **(6)** | **(7)** | **(8)** | **(9)** | **(10)** | **(11)** | **(12)** | **(13)** | **(14)** | **(15)** |  |
| --- | --- | --- | --- | --- | --- | --- | --- | --- | --- | --- | --- | --- | --- | --- | --- | --- |
|  | **Wholesale prices (log)** | | | | | | | | | | | | | | | |
|  | **Rice** | **Wheat** | **Gram** | **Tur** | **Urad** | **Moong** | **Masoor** | **Milk** | **Onion** | **Potatoes** | **Tomatoes** | **Packaged Oils** | **Tea** | **Salt** | **Sugar** |  |
| COVID 19 ^a^ (dummy) | 0.025 | 0.034** | 0.023 | -0.006 | 0.032 | 0.034 | 0.090** | 0.034** | -0.606*** | 0.028 | -0.354*** | 0.018** | 0.028* | 0.030* | 0.017 |  |
|  | (0.016) | (0.011) | (0.022) | (0.022) | (0.024) | (0.031) | (0.027) | (0.010) | (0.141) | (0.023) | (0.086) | (0.007) | (0.012) | (0.013) | (0.013) |  |
| Rainfall (log) | 0.002 | 0.001 | 0.002 | 0.002 | 0.003** | 0.008*** | 0.006*** | 0.002 | -0.004 | -0.006 | 0.007 | 0.002* | 0.002 | 0.003 | 0.002* |  |
|  | (0.001) | (0.002) | (0.001) | (0.002) | (0.001) | (0.001) | (0.001) | (0.001) | (0.011) | (0.005) | (0.007) | (0.001) | (0.001) | (0.002) | (0.001) |  |
| Year fixed effects | Yes | Yes | Yes | Yes | Yes | Yes | Yes | Yes | Yes | Yes | Yes | Yes | Yes | Yes | Yes |  |
| Market fixed effects | Yes | Yes | Yes | Yes | Yes | Yes | Yes | Yes | Yes | Yes | Yes | Yes | Yes | Yes | Yes |  |
| Group-specific time trend | Yes | Yes | Yes | Yes | Yes | Yes | Yes | Yes | Yes | Yes | Yes | Yes | Yes | Yes | Yes |  |
| Monthly time dummies | Yes | Yes | Yes | Yes | Yes | Yes | Yes | Yes | Yes | Yes | Yes | Yes | Yes | Yes | Yes |  |
| State x monthly time dummies | Yes | Yes | Yes | Yes | Yes | Yes | Yes | Yes | Yes | Yes | Yes | Yes | Yes | Yes | Yes |  |
| Observations | 1,954 | 1,797 | 1,945 | 1,926 | 1,947 | 1,954 | 1,940 | 1,434 | 1,954 | 1,953 | 1,943 | 1,947 | 1,667 | 1,910 | 1,954 |  |
| R-squared | 0.952 | 0.953 | 0.858 | 0.853 | 0.916 | 0.906 | 0.894 | 0.962 | 0.910 | 0.923 | 0.799 | 0.924 | 0.971 | 0.976 | 0.872 |  |

Regressions are conducted for the period Jan 2019 to June 2020. ^a^ Daily COVID 19 caseloads cross 100 for a specific state in which market i is located. * Significant at 10% level, ** Significant at 5% level, ***Significant at 1% level. Nominal prices series have been deflated by the wholesale price index (2011-12 prices) and then all prices are log-transformed. Standard errors are clustered by quarters of the year in parenthesis. Pulses: Gram (Chickpea), Tur (Pigeon pea), Urad (Black gram), Moong (Yellow lentils), Masoor (Red lentils).

**Table A20: Absolute price difference between retail and wholesale prices (robustness check: standard errors clustered by quarters of the year)**

|  | **(1)** | **(2)** | **(3)** | **(4)** | **(5)** | **(6)** | **(7)** | **(8)** | **(9)** | **(10)** | **(11)** | **(12)** | **(13)** | **(14)** | **(15)** |
| --- | --- | --- | --- | --- | --- | --- | --- | --- | --- | --- | --- | --- | --- | --- | --- |
|  | **Absolute price difference between retail and wholesale prices (log)** | | | | | | | | | | | | | | |
|  | **Rice** | **Wheat** | **Gram** | **Tur** | **Urad** | **Moong** | **Masoor** | **Milk** | **Onion** | **Potatoes** | **Tomatoes** | **Packaged Oils** | **Tea** | **Salt** | **Sugar** |
| COVID-19 ^a^ (dummy) | -0.012 | -0.019 | 0.103** | 0.119 | 0.091 | 0.148** | 0.194*** | -0.047** | -0.253** | 0.111* | -0.101*** | 0.059 | 0.115** | 0.066** | 0.050 |
|  | (0.020) | (0.024) | (0.037) | (0.059) | (0.065) | (0.047) | (0.040) | (0.017) | (0.069) | (0.051) | (0.022) | (0.033) | (0.042) | (0.019) | (0.036) |
| Rain (log) | -0.004 | 0.001 | 0.002 | 0.000 | -0.010 | -0.012 | 0.002 | 0.006 | -0.002 | -0.009** | -0.025*** | -0.003 | -0.001 | 0.003 | -0.002 |
|  | (0.003) | (0.007) | (0.006) | (0.006) | (0.007) | (0.008) | (0.007) | (0.008) | (0.007) | (0.003) | (0.004) | (0.008) | (0.010) | (0.002) | (0.006) |
| Year fixed effects | Yes | Yes | Yes | Yes | Yes | Yes | Yes | Yes | Yes | Yes | Yes | Yes | Yes | Yes | Yes |
| Market fixed effects | Yes | Yes | Yes | Yes | Yes | Yes | Yes | Yes | Yes | Yes | Yes | Yes | Yes | Yes | Yes |
| Group-specific time trend | Yes | Yes | Yes | Yes | Yes | Yes | Yes | Yes | Yes | Yes | Yes | Yes | Yes | Yes | Yes |
| Monthly time dummies | Yes | Yes | Yes | Yes | Yes | Yes | Yes | Yes | Yes | Yes | Yes | Yes | Yes | Yes | Yes |
| State x monthly time dummies | Yes | Yes | Yes | Yes | Yes | Yes | Yes | Yes | Yes | Yes | Yes | Yes | Yes | Yes | Yes |
| Observations | 1,954 | 1,796 | 1,944 | 1,926 | 1,946 | 1,954 | 1,937 | 1,432 | 1,954 | 1,953 | 1,943 | 1,947 | 1,666 | 1,910 | 1,954 |
| R-squared | 0.829 | 0.806 | 0.871 | 0.850 | 0.825 | 0.831 | 0.813 | 0.872 | 0.800 | 0.795 | 0.789 | 0.863 | 0.864 | 0.849 | 0.790 |

Regressions are conducted for the period Jan 2019 to June 2020. ^a^ Daily COVID 19 caseloads cross 100 for a specific state in which market i is located.* Significant at 10% level, ** Significant at 5% level, ***Significant at 1% level. Nominal prices series have been deflated by the wholesale price index (2011-12 prices). Dependent variable is measured as absolute price difference between retail and wholesale prices (Rs per unit). Standard errors are clustered by quarters of the year in parenthesis. Pulses: Gram (Chickpea), Tur (Pigeon pea), Urad (Black gram), Moong (Yellow lentils), Masoor (Red lentils).

**Table A21: Spread of COVID-19 and spatial retail price difference (robustness check: standard errors clustered by quarters of the year)**

|  | **(1)** | **(2)** | **(3)** | **(4)** | **(5)** | **(6)** | **(7)** | **(8)** | **(9)** | **(10)** | **(11)** | **(12)** | **(13)** | **(14)** | **(15)** |
| --- | --- | --- | --- | --- | --- | --- | --- | --- | --- | --- | --- | --- | --- | --- | --- |
|  | **Absolute retail price difference between market j and k (log)** | | | | | | | | | | | | | | |
|  | **Rice** | **Wheat** | **Gram** | **Tur** | **Urad** | **Moong** | **Masoor** | **Milk** | **Onion** | **Potatoes** | **Tomatoes** | **Packaged Oils** | **Tea** | **Salt** | **Sugar** |
| COVID-19 ^a^ (dummy) | 0.023 | 0.125* | 0.517*** | 0.179** | -0.006 | 0.296** | 0.313*** | 0.032 | -0.147 | 0.149 | 0.306** | 0.102** | 0.026 | 0.031 | 0.020 |
|  | (0.013) | (0.050) | (0.089) | (0.052) | (0.049) | (0.088) | (0.061) | (0.035) | (0.092) | (0.098) | (0.078) | (0.029) | (0.020) | (0.064) | (0.021) |
| Rain (log) | 0.000 | -0.010 | -0.031 | 0.010 | -0.017 | -0.016 | 0.035* | -0.026 | 0.049* | -0.010 | 0.008 | 0.014 | 0.011 | 0.002 | -0.005 |
|  | (0.019) | (0.014) | (0.026) | (0.025) | (0.019) | (0.013) | (0.016) | (0.016) | (0.021) | (0.008) | (0.015) | (0.012) | (0.015) | (0.008) | (0.013) |
| Monthly time dummies | Yes | Yes | Yes | Yes | Yes | Yes | Yes | Yes | Yes | Yes | Yes | Yes | Yes | Yes | Yes |
| Group specific trend | Yes | Yes | No | No | Yes | Yes | Yes | No | No | Yes | No | Yes | No | Yes | Yes |
| Common time trend | No | No | Yes | Yes | No | No | No | Yes | Yes | No | Yes | No | No | No | No |
| Quadratic time trend | No | No | Yes | Yes | No | No | No | Yes | No | No | Yes | No | No | No | No |
| Year fixed effects | Yes | Yes | Yes | Yes | Yes | Yes | Yes | Yes | Yes | Yes | Yes | Yes | Yes | Yes | Yes |
| Market pair fixed effects | Yes | Yes | Yes | Yes | Yes | Yes | Yes | Yes | Yes | Yes | Yes | Yes | Yes | Yes | Yes |
| Observations | 10,868 | 7,842 | 10,547 | 10,200 | 10,867 | 11,046 | 11,001 | 10,655 | 11,059 | 11,083 | 11,031 | 11,157 | 10,503 | 10,211 | 10827 |
| R-squared | 0.785 | 0.754 | 0.524 | 0.353 | 0.607 | 0.565 | 0.632 | 0.745 | 0.387 | 0.542 | 0.335 | 0.705 | 0.804 | 0.836 | 0.724 |

Regressions are conducted for the period Jan 20019 to June 2020. Dependent variable is the log transformed absolute price difference between market *i* and market *j.* ^a^ COVID-19 takes a value of 1 if at least one market crosses the 100-caseload threshold. * Significant at 10% level, ** Significant at 5% level, ***Significant at 1% level. Nominal prices series have been deflated by the wholesale price index (2011-12 prices) and then all prices are log-transformed. Pulses: Gram (Chickpea), Tur (Pigeon pea), Urad (Black gram), Moong (Yellow lentils), Masoor (Red lentils).

**Table A22: Spread of COVID-19 and spatial wholesale price difference (robustness check: standard errors clustered by quarters of the year)**

|  | **(1)** | **(2)** | **(3)** | **(4)** | **(5)** | **(6)** | **(7)** | **(8)** | **(9)** | **(10)** | **(11)** | **(12)** | **(13)** | **(14)** | **(15)** |
| --- | --- | --- | --- | --- | --- | --- | --- | --- | --- | --- | --- | --- | --- | --- | --- |
|  | **Absolute wholesale price difference between market j and k (log)** | | | | | | | | | | | | | | |
|  | **Rice** | **Wheat** | **Gram** | **Tur** | **Urad** | **Moong** | **Masoor** | **Milk** | **Onion** | **Potatoes** | **Tomatoes** | **Packaged Oils** | **Tea** | **Salt** | **Sugar** |
| COVID-19 ^a^ (dummy) | 0.024 | 0.021 | 0.368** | 0.201*** | -0.113 | 0.161 | 0.377*** | 0.036 | -0.008 | 0.160** | 0.140 | -0.036 | 0.110* | 0.051 | 0.026 |
|  | (0.033) | (0.048) | (0.106) | (0.049) | (0.066) | (0.098) | (0.087) | (0.038) | (0.172) | (0.040) | (0.095) | (0.045) | (0.044) | (0.039) | (0.042) |
| Rain (log) | -0.007 | -0.037* | -0.033* | -0.003 | 0.000 | -0.006 | 0.027 | -0.031 | 0.041** | -0.010 | 0.000 | 0.006 | -0.014 | -0.005 | -0.002 |
|  | (0.011) | (0.016) | (0.013) | (0.017) | (0.016) | (0.015) | (0.017) | (0.027) | (0.013) | (0.027) | (0.029) | (0.011) | (0.010) | (0.010) | (0.013) |
| Monthly time dummies | Yes | Yes | Yes | Yes | Yes | Yes | Yes | Yes | Yes | Yes | Yes | Yes | Yes | Yes | Yes |
| Group specific trend | Yes | Yes | Yes | No | Yes | Yes | Yes | No | No | No | No | Yes | Yes | No | No |
| Common time trend | No | No | No | Yes | No | No | No | Yes | Yes | Yes | Yes | No | No | Yes | Yes |
| Quadratic time trend | No | No | No | Yes | No | No | No | Yes | Yes | Yes | Yes | No | No | No | Yes |
| Year fixed effects | Yes | Yes | Yes | Yes | Yes | Yes | Yes | Yes | Yes | Yes | Yes | Yes | Yes | Yes | Yes |
| Market pair fixed effects | Yes | Yes | Yes | Yes | Yes | Yes | Yes | Yes | Yes | Yes | Yes | Yes | Yes | Yes | Yes |
| Observations | 10,459 | 7,437 | 10,114 | 9,694 | 10,345 | 10,494 | 10,463 | 5,760 | 10,529 | 10,540 | 10,483 | 10,529 | 7,150 | 10,410 | 10,490 |
| R-squared | 0.789 | 0.746 | 0.611 | 0.314 | 0.616 | 0.603 | 0.680 | 0.728 | 0.448 | 0.503 | 0.401 | 0.656 | 0.909 | 0.847 | 0.645 |

Regressions are conducted for the period Jan 20019 to June 2020. Dependent variable is the log transformed absolute price difference between market *i* and market *j.* ^a^ COVID-19 takes a value of 1 if at least one market crosses the 100-caseload threshold. * Significant at 10% level, ** Significant at 5% level, ***Significant at 1% level. Nominal prices series have been deflated by the wholesale price index (2011-12 prices) and then all prices are log-transformed. Pulses: Gram (Chickpea), Tur (Pigeon pea), Urad (Black gram), Moong (Yellow lentils), Masoor (Red lentils).

**Table A23: Spread of COVID-19 and market prices (cut-off 300 caseloads))**

|  |  | **(1)** | **(2)** | **(3)** |
| --- | --- | --- | --- | --- |
|  |  | **Retail prices** | **Wholesale prices** | **Vertical spread (retail-wholesale)** |
|  | Rice | 0.067*** | 0.085*** | -0.052 |
| **Storable** | Wheat | 0.026** | 0.036*** | -0.015 |
|  | Gram | 0.029** | 0.017 | 0.109** |
|  | Tur | 0.018 | 0.007 | 0.127** |
|  | Urad | 0.049* | 0.034 | 0.153** |
|  | Moong | 0.061*** | 0.047*** | 0.167** |
|  | Masoor | 0.096*** | 0.074*** | 0.253*** |
|  | Packaged Oils | 0.012 | 0.012 | 0.030 |
|  | Tea | 0.015 | 0.016 | 0.092 |
|  | Salt | 0.041** | 0.019 | 0.092** |
|  | Sugar | 0.018** | 0.013* | 0.059 |
| **Perishable** | Milk | 0.015* | 0.033*** | -0.092* |
|  | Onion | -0.414*** | -0.564*** | -0.183*** |
|  | Potatoes | 0.072*** | 0.027 | 0.130** |
|  | Tomatoes | -0.177*** | -0.239*** | -0.081 |

Table 3 shows the coefficient of the term ${COVID19}_{ist}$ in equation 1. In column (1) the outcome variable is retail prices (log) and in column (2) the outcome variable is wholesale prices (log) and in column (3) the outcome variable is spread in retail and wholesale prices. We run separate regressions for each of the 15 commodities. the Regressions are conducted for the period Jan 2019 to June 2020. *Significant at 10% level. ** Significant at 5% level, ***Significant at 1% level. For brevity we do not show the full regression models. Nominal prices series have been deflated by the wholesale price index (2011-12 prices) and then all prices are log-transformed. Pulses: Gram (Chickpea), Tur (Pigeon pea), Urad (Black gram), Moong (Yellow lentils), Masoor (Red lentils).

**Table A24: Estimates on retail prices (robustness check: continuous COVID-19 variable)**

|  | **(1)** | **(2)** | **(3)** | **(4)** | **(5)** | **(6)** | **(7)** | **(8)** | **(9)** | **(10)** | **(11)** | **(12)** | **(13)** | **(14)** | **(15)** |  |
| --- | --- | --- | --- | --- | --- | --- | --- | --- | --- | --- | --- | --- | --- | --- | --- | --- |
|  | **Retail prices (log)** | | | | | | | | | | | | | | | |
|  | **Rice** | **Wheat** | **Gram** | **Tur** | **Urad** | **Moong** | **Masoor** | **Milk** | **Onion** | **Potatoes** | **Tomatoes** | **Packaged oils** | **Tea** | **Salt** | **Sugar** |  |
| COVID-19 caseloads (IHS^a^) | 0.010*** | 0.010*** | 0.018*** | 0.010*** | 0.017*** | 0.018*** | 0.025*** | 0.007** | -0.112*** | 0.025*** | -0.051*** | 0.004** | 0.008*** | 0.011*** | 0.012*** |  |
|  | (0.002) | (0.002) | (0.003) | (0.003) | (0.004) | (0.003) | (0.003) | (0.003) | (0.012) | (0.006) | (0.009) | (0.002) | (0.002) | (0.003) | (0.001) |  |
| Night light (log) | 0.040** | 0.031 | -0.007 | -0.005 | 0.026 | 0.007 | 0.013 | -0.004 | -0.016 | 0.051* | -0.045 | 0.021** | 0.006 | -0.006 | 0.012* |  |
|  | (0.016) | (0.019) | (0.012) | (0.014) | (0.024) | (0.014) | (0.017) | (0.009) | (0.039) | (0.028) | (0.072) | (0.010) | (0.015) | (0.020) | (0.007) |  |
| Rainfall (log) | 0.001 | 0.001 | -0.001 | 0.001 | 0.001 | 0.003 | 0.002 | 0.000 | 0.006 | -0.008** | 0.005 | 0.001 | 0.000 | 0.001 | 0.000 |  |
|  | (0.001) | (0.002) | (0.002) | (0.002) | (0.002) | (0.002) | (0.002) | (0.002) | (0.006) | (0.003) | (0.006) | (0.001) | (0.001) | (0.002) | (0.001) |  |
| Diesel price (log) | -0.206*** | -0.196** | -0.328*** | -0.452*** | -0.357*** | -0.281*** | 0.035 | -0.027 | -0.468* | -0.641*** | -1.220*** | -0.004 | -0.033 | -0.037 | -0.215*** |  |
|  | (0.065) | (0.081) | (0.069) | (0.083) | (0.099) | (0.097) | (0.081) | (0.057) | (0.266) | (0.163) | (0.302) | (0.044) | (0.048) | (0.081) | (0.036) |  |
| Year fixed effects | Yes | Yes | Yes | Yes | Yes | Yes | Yes | Yes | Yes | Yes | Yes | Yes | Yes | Yes | Yes |  |
| Market fixed effects | Yes | Yes | Yes | Yes | Yes | Yes | Yes | Yes | Yes | Yes | Yes | Yes | Yes | Yes | Yes |  |
| Group-specific time trend | Yes | Yes | Yes | Yes | Yes | No | Yes | No | Yes | Yes | Yes | Yes | Yes | Yes | Yes |  |
| Common time trend | No | No | No | No | No | Yes | No | Yes | No | No | No | No | No | No | No |  |
| Monthly time dummies | Yes | Yes | Yes | Yes | Yes | Yes | Yes | Yes | Yes | Yes | Yes | Yes | Yes | Yes | Yes |  |
| State x monthly time dummies | Yes | Yes | Yes | Yes | Yes | Yes | Yes | Yes | Yes | Yes | Yes | Yes | Yes | Yes | Yes |  |
| Observations | 1,971 | 1,824 | 1,961 | 1,946 | 1,966 | 1,971 | 1,966 | 1,964 | 1,971 | 1,971 | 1,967 | 1,971 | 1,956 | 1,971 | 1,971 |  |
| R-squared | 0.702 | 0.601 | 0.507 | 0.757 | 0.846 | 0.786 | 0.739 | 0.376 | 0.918 | 0.797 | 0.671 | 0.773 | 0.512 | 0.562 | 0.527 |  |

^a^ Inverse hyperbolic sine transformation of the average daily COVID-19 caseloads are used as the main indicator for spread of COVID-19. Regressions are conducted for the period Jan 2019 to June 2020. * Significant at 10% level, ** Significant at 5% level, ***Significant at 1% level. Nominal prices series have been deflated by the wholesale price index (2011-12 prices) and then all prices are log-transformed. Standard errors are clustered by markets in parenthesis. Pulses: Gram (Chickpea), Tur (Pigeon pea), Urad (Black gram), Moong (Yellow lentils), Masoor (Red lentils).

**Table A25: Estimates on wholesale prices (robustness check: continuous COVID-19 variable)**

|  | **(1)** | **(2)** | **(3)** | **(4)** | **(5)** | **(6)** | **(7)** | **(8)** | **(9)** | **(10)** | **(11)** | **(12)** | **(13)** | **(14)** | **(15)** |  |
| --- | --- | --- | --- | --- | --- | --- | --- | --- | --- | --- | --- | --- | --- | --- | --- | --- |
|  | **Wholesale prices (log)** | | | | | | | | | | | | | | | |
|  | **Rice** | **Wheat** | **Gram** | **Tur** | **Urad** | **Moong** | **Masoor** | **Milk** | **Onion** | **Potatoes** | **Tomatoes** | **Packaged oils** | **Tea** | **Salt** | **Sugar** |  |
| COVID-19 caseloads (IHS ^a^) | 0.011*** | 0.012*** | 0.015*** | 0.007*** | 0.015*** | 0.017*** | 0.024*** | 0.010*** | -0.123*** | 0.015*** | -0.068*** | 0.004*** | 0.008*** | 0.009*** | 0.010*** |  |
|  | (0.002) | (0.002) | (0.002) | (0.003) | (0.004) | (0.003) | (0.003) | (0.004) | (0.011) | (0.006) | (0.009) | (0.002) | (0.002) | (0.004) | (0.001) |  |
| Night light (log) | 0.044** | 0.047** | -0.016 | -0.004 | 0.037 | 0.005 | 0.012 | 0.002 | -0.017 | 0.042 | -0.074 | 0.022** | 0.000 | -0.013 | 0.004 |  |
|  | (0.017) | (0.018) | (0.012) | (0.016) | (0.026) | (0.015) | (0.015) | (0.011) | (0.047) | (0.029) | (0.091) | (0.011) | (0.009) | (0.021) | (0.006) |  |
| Rainfall (log) | 0.001 | 0.000 | 0.000 | 0.002 | 0.003 | 0.003* | 0.004** | -0.001 | 0.009 | -0.006 | 0.015** | 0.002** | 0.001 | 0.001 | 0.001 |  |
|  | (0.001) | (0.002) | (0.001) | (0.002) | (0.002) | (0.002) | (0.002) | (0.004) | (0.007) | (0.004) | (0.007) | (0.001) | (0.002) | (0.002) | (0.001) |  |
| Diesel price (log) | -0.208*** | -0.199** | -0.280*** | -0.393*** | -0.363*** | -0.260** | 0.046 | -0.043 | -1.043*** | -0.644*** | -1.211*** | -0.007 | 0.005 | -0.028 | -0.215*** |  |
|  | (0.066) | (0.084) | (0.070) | (0.083) | (0.089) | (0.106) | (0.078) | (0.072) | (0.277) | (0.202) | (0.336) | (0.046) | (0.047) | (0.088) | (0.030) |  |
| Year fixed effects | Yes | Yes | Yes | Yes | Yes | Yes | Yes | Yes | Yes | Yes | Yes | Yes | Yes | Yes | Yes |  |
| Market fixed effects | Yes | Yes | Yes | Yes | Yes | Yes | Yes | Yes | Yes | Yes | Yes | Yes | Yes | Yes | Yes |  |
| Group-specific time trend | No | Yes | Yes | Yes | Yes | No | Yes | No | Yes | Yes | Yes | Yes | Yes | Yes | Yes |  |
| Common time trend | No | No | No | No | No | Yes | No | Yes | No | No | No | No | No | No | No |  |
| Monthly time dummies | Yes | Yes | Yes | Yes | Yes | Yes | Yes | Yes | Yes | Yes | Yes | Yes | Yes | Yes | Yes |  |
| State x monthly time dummies | Yes | Yes | Yes | Yes | Yes | Yes | Yes | Yes | Yes | Yes | Yes | Yes | Yes | Yes | Yes |  |
| Observations | 1,954 | 1,797 | 1,945 | 1,926 | 1,947 | 1,954 | 1,940 | 1,434 | 1,954 | 1,953 | 1,943 | 1,947 | 1,667 | 1,910 | 1,954 |  |
| R-squared | 0.716 | 0.603 | 0.499 | 0.740 | 0.853 | 0.772 | 0.773 | 0.374 | 0.916 | 0.822 | 0.686 | 0.776 | 0.616 | 0.622 | 0.552 |  |

^a^Inverse hyperbolic sine transformation of the average daily COVID-19 caseloads are used as the main indicator for spread of COVID-19. Regressions are conducted for the period Jan 2019 to June 2020. * Significant at 10% level, ** Significant at 5% level, ***Significant at 1% level. Nominal prices series have been deflated by the wholesale price index (2011-12 prices) and then all prices are log-transformed. Pulses: Gram (Chickpea), Tur (Pigeon pea), Urad (Black gram), Moong (Yellow lentils), Masoor (Red lentils).

**Table A26: Absolute price difference between retail and wholesale prices (****robustness check: continuous COVID-19 variable)**

|  | **(1)** | **(2)** | **(3)** | **(4)** | **(5)** | **(6)** | **(7)** | **(8)** | **(9)** | **(10)** | **(11)** | **(12)** | **(13)** | **(14)** | **(15)** |
| --- | --- | --- | --- | --- | --- | --- | --- | --- | --- | --- | --- | --- | --- | --- | --- |
|  | **Absolute price difference between retail and wholesale perice (log)** | | | | | | | | | | | | | | |
|  | **Rice** | **Wheat** | **Gram** | **Tur** | **Urad** | **Moong** | **Masoor** | **Milk** | **Onion** | **Potatoes** | **Tomatoes** | **Packaged oils** | **Tea** | **Salt** | **Sugar** |
| COVID-19 caseloads (IHS^a^) | -0.004 | -0.004 | 0.041*** | 0.037*** | 0.026* | 0.029** | 0.041*** | -0.004 | -0.079*** | 0.037*** | -0.007 | 0.014 | 0.018 | 0.012** | 0.018** |
|  | (0.008) | (0.009) | (0.010) | (0.010) | (0.015) | (0.013) | (0.013) | (0.010) | (0.012) | (0.009) | (0.010) | (0.009) | (0.014) | (0.006) | (0.008) |
| Night light (log) | -0.004 | -0.014 | 0.080 | -0.027 | -0.075 | 0.062 | 0.040 | -0.027 | -0.035 | 0.100 | 0.016 | -0.021 | -0.031 | 0.006 | 0.119** |
|  | (0.037) | (0.051) | (0.060) | (0.070) | (0.077) | (0.067) | (0.087) | (0.072) | (0.059) | (0.066) | (0.083) | (0.056) | (0.111) | (0.042) | (0.047) |
| Rainfall (log) | 0.002 | 0.002 | -0.000 | -0.002 | -0.013 | -0.013 | -0.001 | -0.008 | 0.003 | -0.011 | -0.024*** | -0.003 | 0.000 | 0.002 | -0.001 |
|  | (0.005) | (0.006) | (0.008) | (0.007) | (0.009) | (0.008) | (0.009) | (0.010) | (0.008) | (0.008) | (0.008) | (0.008) | (0.010) | (0.004) | (0.005) |
| Diesel price (log) | -0.312 | -0.243 | -0.615* | -0.873** | -0.405 | -0.038 | -0.015 | 0.179 | 1.136*** | -0.522* | -0.628* | -0.328 | -0.858** | 0.028 | -0.230 |
|  | (0.199) | (0.252) | (0.336) | (0.349) | (0.351) | (0.383) | (0.329) | (0.298) | (0.343) | (0.301) | (0.377) | (0.264) | (0.432) | (0.220) | (0.253) |
| Year fixed effects | Yes | Yes | Yes | Yes | Yes | Yes | Yes | Yes | Yes | Yes | Yes | Yes | Yes | Yes | Yes |
| Market fixed effects | Yes | Yes | Yes | Yes | Yes | Yes | Yes | Yes | Yes | Yes | Yes | Yes | Yes | Yes | Yes |
| Group-specific time trend | Yes | Yes | Yes | Yes | Yes | Yes | Yes | Yes | Yes | Yes | Yes | Yes | Yes | Yes | Yes |
| Monthly time dummies | Yes | Yes | Yes | Yes | Yes | Yes | Yes | Yes | Yes | Yes | Yes | Yes | Yes | Yes | Yes |
| State x monthly time dummies | Yes | Yes | Yes | Yes | Yes | Yes | Yes | Yes | Yes | Yes | Yes | Yes | Yes | Yes | Yes |
| Observations | 1,954 | 1,796 | 1,944 | 1,926 | 1,946 | 1,954 | 1,937 | 1,432 | 1,954 | 1,953 | 1,943 | 1,947 | 1,666 | 1,910 | 1,954 |
| R-squared | 0.138 | 0.475 | 0.542 | 0.548 | 0.501 | 0.526 | 0.427 | 0.118 | 0.673 | 0.470 | 0.447 | 0.424 | 0.550 | 0.533 | 0.435 |

^a^Inverse hyperbolic sine transformation of the average daily COVID-19 caseloads are used as the main indicator for spread of COVID-19. Regressions are conducted for the period Jan 2019 to June 2020. ^*^ Daily COVID 19 caseloads cross 100 for a specific state in which market i is located. * Significant at 10% level, ** Significant at 5% level, ***Significant at 1% level. Nominal prices series have been deflated by the wholesale price index (2011-12 prices). Dependent variable is measured as absolute price difference between retail and wholesale prices (Rs per unit). Pulses: Gram (Chickpea), Tur (Pigeon pea), Urad (Black gram), Moong (Yellow lentils), Masoor (Red lentils).

**Table A27:** **Spread of COVID-19 and spatial retail price difference (robustness check: continuous COVID-19 variable)**

|  | **(1)** | **(2)** | **(3)** | **(4)** | **(5)** | **(6)** | **(7)** | **(8)** | **(9)** | **(10)** | **(11)** | **(12)** | **(13)** | **(14)** | **(15)** |  |
| --- | --- | --- | --- | --- | --- | --- | --- | --- | --- | --- | --- | --- | --- | --- | --- | --- |
|  | **Absolute retail price difference between market j and k (log)** | | | | | | | | | | | | | | | |
|  | **Rice** | **Wheat** | **Gram** | **Tur** | **Urad** | **Moong** | **Masoor** | **Milk** | **Onion** | **Potatoes** | **Tomatoes** | **Packaged oils** | **Tea** | **Salt** | **Sugar** |  |
| COVID-19 caseloads (IHS^a^) | 0.008 | 0.025** | 0.089*** | 0.107*** | -0.013 | 0.094*** | 0.084*** | 0.019** | -0.031** | 0.052*** | 0.071*** | 0.023** | 0.015* | 0.001 | -0.003 |  |
|  | (0.009) | (0.010) | (0.013) | (0.022) | (0.011) | (0.012) | (0.011) | (0.009) | (0.013) | (0.010) | (0.014) | (0.009) | (0.009) | (0.007) | (0.009) |  |
| Rainfall (log) | -0.000 | -0.012 | -0.034*** | 0.007 | -0.015 | -0.023** | 0.028*** | -0.027*** | 0.044*** | -0.014 | 0.005 | 0.012 | 0.011** | -0.004 | -0.004 |  |
|  | (0.007) | (0.010) | (0.009) | (0.010) | (0.012) | (0.010) | (0.010) | (0.007) | (0.012) | (0.010) | (0.011) | (0.008) | (0.005) | (0.005) | (0.008) |  |
| Monthly time dummies | Yes | Yes | Yes | Yes | Yes | Yes | Yes | Yes | Yes | Yes | Yes | Yes | Yes | Yes | Yes |  |
| Group specific trend | Yes | Yes | No | No | Yes | Yes | Yes | No | Yes | Yes | No | Yes | No | No | Yes |  |
| Common time trend | No | No | Yes | Yes | No | No | No | Yes | No | No | Yes | No | Yes | No | No |  |
| Quadratic time trend | No | No | Yes | Yes | No | No | No | Yes | No | No | Yes | No | Yes | No | No |  |
| Year fixed effects | Yes | Yes | Yes | Yes | Yes | Yes | Yes | Yes | Yes | Yes | Yes | Yes | Yes | Yes | Yes |  |
| Market pair fixed effects | Yes | Yes | Yes | Yes | Yes | Yes | Yes | Yes | Yes | Yes | Yes | Yes | Yes | Yes | Yes |  |
| Observations | 10,868 | 7,842 | 10,547 | 10,200 | 10,867 | 11,046 | 11,001 | 10,655 | 11,059 | 11,083 | 11,031 | 11,157 | 10,503 | 10,211 | 10,827 |  |
| R-squared | 0.324 | 0.374 | 0.023 | 0.035 | 0.345 | 0.243 | 0.249 | 0.026 | 0.236 | 0.145 | 0.043 | 0.300 | 0.004 | 0.020 | 0.212 |  |

^a^Inverse hyperbolic sine transformation of the average daily COVID-19 caseloads are used as the main indicator for spread of COVID-19. Regressions are conducted for the period Jan 20019 to June 2020. Dependent variable is the log transformed absolute price difference between market *i* and market *j.** Significant at 10% level, ** Significant at 5% level, ***Significant at 1% level. Nominal prices series have been deflated by the wholesale price index (2011-12 prices) and then all prices are log-transformed. Pulses: Gram (Chickpea), Tur (Pigeon pea), Urad (Black gram), Moong (Yellow lentils), Masoor (Red lentils).

**Table A28:** **Spread of COVID-19 and spatial wholesale price difference (robustness check: continuous COVID-19 variable)**

|  | **(1)** | **(2)** | **(3)** | **(4)** | **(5)** | **(6)** | **(7)** | **(8)** | **(9)** | **(10)** | **(11)** | **(12)** | **(13)** | **(14)** | **(15)** |  |
| --- | --- | --- | --- | --- | --- | --- | --- | --- | --- | --- | --- | --- | --- | --- | --- | --- |
|  | **Absolute wholesale price difference between market j and k (log)** | | | | | | | | | | | | | | | |
|  | **Rice** | **Wheat** | **Gram** | **Tur** | **Urad** | **Moong** | **Masoor** | **Milk** | **Onion** | **Potatoes** | **Tomatoes** | **Packaged oils** | **Tea** | **Salt** | **Sugar** |  |
| COVID-19 caseloads (IHS^a^) | 0.010 | 0.000 | 0.094*** | 0.081** | -0.045 | 0.066*** | 0.111*** | 0.013 | -0.059 | 0.038* | 0.009 | -0.011 | 0.029*** | 0.006 | 0.003 |  |
|  | (0.007) | (0.010) | (0.020) | (0.028) | (0.042) | (0.015) | (0.019) | (0.011) | (0.049) | (0.018) | (0.030) | (0.008) | (0.006) | (0.011) | (0.011) |  |
| Rainfall (log) | -0.008 | -0.036* | -0.041** | -0.005 | 0.017 | -0.013 | 0.017 | -0.032 | 0.040** | -0.012 | 0.000 | 0.007 | -0.016 | -0.005 | -0.002 |  |
|  | (0.010) | (0.017) | (0.015) | (0.016) | (0.024) | (0.012) | (0.016) | (0.028) | (0.012) | (0.027) | (0.029) | (0.011) | (0.010) | (0.011) | (0.013) |  |
| Monthly time dummies | Yes | Yes | Yes | Yes | Yes | Yes | Yes | Yes | Yes | Yes | Yes | Yes | Yes | Yes | Yes |  |
| Group specific trend | Yes | Yes | Yes | No | No | Yes | Yes | No | No | No | No | Yes | Yes | No | No |  |
| Common time trend | No | No | No | Yes | Yes | No | No | Yes | Yes | Yes | Yes | No | No | Yes | Yes |  |
| Quadratic time trend | No | No | No | Yes | Yes | No | No | Yes | Yes | Yes | Yes | No | No | No | Yes |  |
| Year fixed effects | Yes | Yes | Yes | Yes | Yes | Yes | Yes | Yes | Yes | Yes | Yes | Yes | Yes | Yes | Yes |  |
| Market pair fixed effects | Yes | Yes | Yes | Yes | Yes | Yes | Yes | Yes | Yes | Yes | Yes | Yes | Yes | Yes | Yes |  |
| Observations | 10,459 | 7,437 | 10,114 | 9,694 | 10,345 | 10,494 | 10,463 | 5,760 | 10,529 | 10,540 | 10,483 | 10,529 | 7,150 | 10,410 | 10,490 |  |
| R-squared | 0.789 | 0.746 | 0.613 | 0.315 | 0.461 | 0.605 | 0.683 | 0.728 | 0.493 | 0.503 | 0.400 | 0.656 | 0.909 | 0.847 | 0.645 |  |

^a^Inverse hyperbolic sine transformation of the average daily COVID-19 caseloads are used as the main indicator for spread of COVID-19. Regressions are conducted for the period Jan 20019 to June 2020. Dependent variable is the log transformed absolute price difference between market *j* and market k*.** Significant at 10% level, ** Significant at 5% level, ***Significant at 1% level. Nominal prices series have been deflated by the wholesale price index (2011-12 prices) and then all prices are log-transformed. Pulses: Gram (Chickpea), Tur (Pigeon pea), Urad (Black gram), Moong (Yellow lentils), Masoor (Red lentils).

**Table A29: National lockdown and retail prices**

|  | **(1)** | **(2)** | **(3)** | **(4)** | **(5)** | **(6)** | **(7)** | **(8)** | **(9)** | **(10)** | **(11)** | **(12)** | **(13)** | **(14)** | **(15)** |  |
| --- | --- | --- | --- | --- | --- | --- | --- | --- | --- | --- | --- | --- | --- | --- | --- | --- |
|  | **Retail prices (log)** | | | | | | | | | | | | | | | |
|  | **Rice** | **Wheat** | **Gram** | **Tur** | **Urad** | **Moong** | **Masoor** | **Milk** | **Onion** | **Potatoes** | **Tomatoes** | **Packaged Oils** | **Tea** | **Salt** | **Sugar** |  |
| After lockdown (dummy) | 0.026 | 0.068* | 0.136*** | 0.144*** | 0.105** | 0.149*** | 0.075* | 0.038 | -0.284*** | 0.277*** | -0.510*** | 0.010 | 0.060** | 0.060* | 0.053*** |  |
|  | (0.023) | (0.035) | (0.030) | (0.041) | (0.048) | (0.044) | (0.040) | (0.024) | (0.061) | (0.071) | (0.082) | (0.010) | (0.028) | (0.032) | (0.014) |  |
| Post (dummy) | -0.373* | 0.729 | 0.469 | 0.315 | 0.508 | 0.804 | -0.088 | 0.018 | 0.848 | 0.286 | -3.010*** | -0.097 | 0.627** | 0.393 | -0.095 |  |
|  | (0.218) | (0.443) | (0.312) | (0.513) | (0.582) | (0.507) | (0.507) | (0.298) | (0.553) | (0.810) | (0.519) | (0.111) | (0.316) | (0.423) | (0.166) |  |
| Night light (log) | 0.031* | 0.032 | -0.015 | -0.003 | 0.017 | 0.003 | -0.006 | -0.003 | 0.086*** | 0.014 | -0.040 | 0.007 | 0.001 | -0.019 | 0.004 |  |
|  | (0.017) | (0.020) | (0.011) | (0.014) | (0.021) | (0.014) | (0.014) | (0.009) | (0.028) | (0.027) | (0.046) | (0.006) | (0.015) | (0.019) | (0.007) |  |
| Rainfall (log) | -0.001 | 0.002 | -0.001 | 0.001 | 0.001 | 0.003* | 0.004*** | 0.001 | 0.011** | -0.009** | 0.008 | 0.000 | 0.000 | 0.001 | -0.000 |  |
|  | (0.001) | (0.003) | (0.002) | (0.002) | (0.002) | (0.002) | (0.001) | (0.001) | (0.004) | (0.004) | (0.006) | (0.001) | (0.001) | (0.002) | (0.001) |  |
| Diesel price (log) | -0.649*** | 0.098 | -0.168 | -0.281 | -0.216 | 0.088 | 0.063 | -0.110 | -0.678 | 0.101 |  | -0.151 | 0.198 | 0.170 | -0.330*** |  |
|  | (0.217) | (0.260) | (0.165) | (0.308) | (0.337) | (0.303) | (0.288) | (0.168) | (0.505) | (0.449) |  | (0.107) | (0.166) | (0.248) | (0.090) |  |
| Month of the year fixed effects | Yes | Yes | Yes | Yes | Yes | Yes | Yes | Yes | Yes | Yes | Yes | Yes | Yes | Yes | Yes |  |
| Market fixed effects | Yes | Yes | Yes | Yes | Yes | Yes | Yes | Yes | Yes | Yes | Yes | Yes | Yes | Yes | Yes |  |
| Common time trend | Yes | Yes | No | No | No | No | Yes | No | No | Yes | No | No | No | Yes | Yes |  |
| Group Specific time trend | No | No | Yes | Yes | Yes | Yes | No | Yes | Yes | No | Yes | Yes | Yes | No | No |  |
| State x monthly time dummies | No | Yes | Yes | Yes | Yes | Yes | Yes | Yes | No | Yes | No | No | Yes | Yes | Yes |  |
| Observations | 1,971 | 1,824 | 1,961 | 1,946 | 1,966 | 1,971 | 1,966 | 1,964 | 1,971 | 1,971 | 1,967 | 1,971 | 1,956 | 1,971 | 1,971 |  |
| R-squared | 0.217 | 0.270 | 0.553 | 0.775 | 0.853 | 0.883 | 0.602 | 0.782 | 0.931 | 0.690 | 0.628 | 0.742 | 0.515 | 0.182 | 0.367 |  |

Regressions are conducted for the period Jan 2019 to June 2020. India went into a national lockdown from the 24th of March 2020. After lockdown takes a value of 1 for April, May and June 2020. * Significant at 10% level, ** Significant at 5% level, ***Significant at 1% level. Nominal prices series have been deflated by the wholesale price index (2011-12 prices). Standard errors are clustered by markets in parenthesis. Pulses: Gram (Chickpea), Tur (Pigeon pea), Urad (Black gram), Moong (Yellow lentils), Masoor (Red lentils).

**Table A30: National lockdown and wholesale prices**

|  | **(1)** | **(2)** | **(3)** | **(4)** | **(5)** | **(6)** | **(7)** | **(8)** | **(9)** | **(10)** | **(11)** | **(12)** | **(13)** | **(14)** | **(15)** |
| --- | --- | --- | --- | --- | --- | --- | --- | --- | --- | --- | --- | --- | --- | --- | --- |
|  | **Wholesale prices (log)** | | | | | | | | | | | | | | |
|  | **Rice** | **Wheat** | **Gram** | **Tur** | **Urad** | **Moong** | **Masoor** | **Milk** | **Onion** | **Potatoes** | **Tomatoes** | **Packaged Oils** | **Tea** | **Salt** | **Sugar** |
| After lockdown (dummy) | 0.047* | 0.090** | 0.123*** | 0.138*** | 0.108** | 0.169*** | 0.091** | 0.058* | -0.351*** | 0.234** | -0.421*** | -0.004 | 0.024 | 0.027 | 0.072*** |
|  | (0.024) | (0.037) | (0.032) | (0.044) | (0.047) | (0.048) | (0.042) | (0.034) | (0.078) | (0.096) | (0.145) | (0.011) | (0.031) | (0.029) | (0.017) |
| Post (dummy) | -0.192 | 0.846* | 0.332 | 0.378 | 0.452 | 1.160** | 0.136 | 0.296 | 1.049 | 0.215 | -7.567*** | -0.185* | 0.187 | 0.158 | 0.264 |
|  | (0.231) | (0.477) | (0.323) | (0.545) | (0.556) | (0.522) | (0.560) | (0.345) | (0.719) | (1.084) | (1.572) | (0.106) | (0.254) | (0.387) | (0.196) |
| Night light (log) | 0.027 | 0.037* | -0.022* | -0.001 | 0.031 | 0.006 | -0.003 | -0.005 | 0.079** | 0.009 | -0.035 | 0.004 | -0.005 | -0.009 | -0.001 |
|  | (0.019) | (0.020) | (0.012) | (0.016) | (0.024) | (0.014) | (0.014) | (0.010) | (0.032) | (0.030) | (0.080) | (0.007) | (0.009) | (0.023) | (0.007) |
| Rainfall (log) | -0.001 | 0.000 | -0.000 | 0.002 | 0.003 | 0.005*** | 0.004** | 0.001 | 0.013** | -0.005 | 0.016** | 0.001 | 0.001 | 0.001 | 0.000 |
|  | (0.001) | (0.003) | (0.002) | (0.002) | (0.002) | (0.002) | (0.002) | (0.002) | (0.005) | (0.006) | (0.007) | (0.001) | (0.002) | (0.002) | (0.001) |
| Diesel price (log) | -0.495** | 0.116 | -0.226 | -0.151 | -0.230 | 0.282 | 0.261 | 0.045 | -0.641 | 0.539 | -3.625*** | -0.190* | 0.081 | 0.090 | -0.104 |
|  | (0.235) | (0.275) | (0.164) | (0.319) | (0.309) | (0.307) | (0.326) | (0.198) | (0.650) | (0.587) | (0.925) | (0.099) | (0.120) | (0.222) | (0.105) |
| Month of the year fixed effects | Yes | Yes | Yes | Yes | Yes | Yes | Yes | Yes | Yes | Yes | Yes | Yes | Yes | Yes | Yes |
| Market fixed effects | Yes | Yes | Yes | Yes | Yes | Yes | Yes | Yes | Yes | Yes | Yes | Yes | Yes | Yes | Yes |
| Common time trend | Yes | Yes | No | No | No | No | Yes | No | No | Yes | No | No | No | Yes | Yes |
| Group Specific time trend | No | No | Yes | Yes | Yes | Yes | No | Yes | Yes | No | Yes | Yes | Yes | No | No |
| State x monthly time dummies | No | Yes | Yes | Yes | Yes | Yes | Yes | Yes | No | Yes | Yes | No | Yes | Yes | Yes |
| Observations | 1,954 | 1,797 | 1,945 | 1,926 | 1,947 | 1,954 | 1,940 | 1,434 | 1,954 | 1,953 | 1,943 | 1,947 | 1,667 | 1,910 | 1,954 |
| R-squared | 0.191 | 0.262 | 0.550 | 0.757 | 0.859 | 0.883 | 0.613 | 0.778 | 0.922 | 0.705 | 0.717 | 0.736 | 0.629 | 0.141 | 0.349 |

Regressions are conducted for the period Jan 2019 to June 2020. India went into a national lockdown from the 24th of March 2020. Here After lockdown takes a value of 1 for April, May and June 2020. * Significant at 10% level, ** Significant at 5% level, ***Significant at 1% level. Nominal prices series have been deflated by the wholesale price index (2011-12 prices). Standard errors are clustered by markets in parenthesis. Pulses: Gram (Chickpea), Tur (Pigeon pea), Urad (Black gram), Moong (Yellow lentils), Masoor (Red lentils).

1. <https://thewire.in/covid-19-india-timeline> ,

   <https://indianexpress.com/article/india/covid-19-india-timeline-looking-back-at-pandemic-induced-lockdown-7241583/> [↑](#footnote-ref-1)
